# Supplementary material for: The Life History of 21 Breast Cancers
Source: Cell. 2012 May 25;149(5):994–1007. doi: 10.1016/j.cell.2012.04.023 (PMC3428864; doi:10.1016/j.cell.2012.04.023)
Supplement: Document S1. Article plus Supplemental Information [file mmc2.pdf]

Cell

The Life History of 21 Breast Cancers

Serena Nik-Zainal,[1,19] Peter Van Loo,[1,2,3,19] David C. Wedge,[1,19] Ludmil B. Alexandrov,[1] Christopher D. Greenman,[1,4,5] King Wai Lau,[1] Keiran Raine,[1] David Jones,[1] John Marshall,[1] Manasa Ramakrishna,[1] Adam Shlien,[1] Susanna L. Cooke,[1] Jonathan Hinton,[1] Andrew Menzies,[1] Lucy A. Stebbings,[1] Catherine Leroy,[1] Mingming Jia,[1] Richard Rance,[1] Laura J. Mudie,[1] Stephen J. Gamble,[1] Philip J. Stephens,[1] Stuart McLaren,[1] Patrick S. Tarpey,[1] Elli Papaemmanuil,[1] Helen R. Davies,[1] Ignacio Varela,[1] David J. McBride,[1] Graham R. Bignell,[1] Kenric Leung,[1] Adam P. Butler,[1] Jon W. Teague,[1] Sancha Martin,[1] Goran Jönsson,[6] Odette Mariani,[7] Sandrine Boyault,[8] Penelope Miron,[9] Aquila Fatima,[9] Anita Langerød,[10] Samuel A.J.R. Aparicio,[11,12] Andrew Tutt,[6] Anieta M. Sieuwerts,[13] Åke Borg,[14] Gilles Thomas,[8] Anne Vincent Salomon,[7] Andrea L. Richardson,[9,15] Anne-Lise Børresen-Dale,[10,16] P. Andrew Futreal,[1] Michael R. Stratton,[1] Peter J. Campbell[1,17,18,*] and Breast Cancer Working Group of the International Cancer Genome Consortium

[1]Cancer Genome Project, Wellcome Trust Sanger Institute, Hinxton CB10 1SA, UK
[2]Center for the Biology of Disease, VIB, Herestraat 49 Box 602, B-3000 Leuven, Belgium
[3]Department of Human Genetics, KU Leuven, Herestraat 49 Box 602, B-3000 Leuven, Belgium
[4]Department of Computing, University of East Anglia, Norwich NR4 7TJ, UK
[5]The Genome Analysis Centre, Norwich Research Park, Norwich NR4 7UH, UK
[6]Breakthrough Breast Cancer Research Unit, Kings College, London SE1 9RT, UK
[7]Institut Curie, Department of Tumor Biology, 26 rue d'Ulm, 75248 Paris Cedex 05, France
[8]Universite Lyon 1, INCa-Synergie, Centre Leon Berard, 28 rue Laennec, Lyon Cedex 08, France
[9]Dana-Farber Cancer Institute, 450 Brookline Avenue, Boston, MA 02215, USA
[10]Department of Genetics, Institute for Cancer Research, Oslo University Hospital, The Norwegian Radium Hospital, 0310 Oslo, Norway
[11]Department of Pathology and Laboratory Medicine, University of British Columbia, Vancouver, British Columbia V6T 2B5, Canada
[12]Molecular Oncology, British Columbia Cancer Research Centre, Vancouver, British Columbia V5Z 1L3, Canada
[13]Erasmus Medical Centre, Postbus 2040, 3000 CA Rotterdam, The Netherlands
[14]Department of Oncology, Lund University, BMC C13, SE-221 84 Lund, Sweden
[15]Brigham and Women's Hospital, Harvard Medical School, 75 Francis St, Boston, MA 02115, USA
[16]K. G. Jebsen Center for Breast Cancer Research, Institute for Clinical Medicine, Faculty of Medicine, University of Oslo, Oslo N-0310, Norway
[17]Department of Haematology, Addenbrooke's Hospital, Cambridge CB2 0QQ, UK
[18]Department of Haematology, University of Cambridge, Cambridge CB2 2XY, UK
[19]These authors contributed equally to this work
*Correspondence: pc8@sanger.ac.uk
DOI 10.1016/j.cell.2012.04.023

SUMMARY

Cancer evolves dynamically as clonal expansions supersede one another driven by shifting selective pressures, mutational processes, and disrupted cancer genes. These processes mark the genome, such that a cancer's life history is encrypted in the somatic mutations present. We developed algorithms to decipher this narrative and applied them to 21 breast cancers. Mutational processes evolve across a cancer's lifespan, with many emerging late but contributing extensive genetic variation. Subclonal diversification is prominent, and most mutations are found in just a fraction of tumor cells. Every tumor has a dominant subclonal lineage, representing more than 50% of tumor cells. Minimal expansion of these subclones occurs until many hundreds to thousands of mutations have accumulated, implying the existence of long-lived, quiescent cell lineages capable of substantial proliferation upon acquisition of enabling genomic changes. Expansion of the dominant subclone to an appreciable mass may therefore represent the final rate-limiting step in a breast cancer's development, triggering diagnosis.

INTRODUCTION

Age-incidence curves of most common epithelial cancers show rapidly increasing rates after the 4th–5th decades of life. Classic mathematical models of tumor development developed by Armitage and Doll (Armitage and Doll, 1954; Hornsby et al., 2007) suggested that 5–8 rate-limiting events are required to generate such incidence patterns. Since these studies were performed in the 1950s, we have learnt much about the biological and genetic basis of cancer. In particular, evolution toward cancer often occurs on a phenotypic spectrum of increasingly disordered premalignant stages, as "hallmark" cellular processes are cumulatively co-opted or ablated in the cancer cells (Hanahan and Weinberg, 2011). Somatic mutation is the fundamental mechanism by which cancer cells suborn these pathways (Stratton et al., 2009), notwithstanding the contributions of epigenetic changes, cues from the local microenvironment and germline genetic variation.

Whole-cancer genomes sequenced to date carry thousands to tens of thousands of somatic mutations (Chapman et al., 2011; Ding et al., 2010; Ley et al., 2010; Pleasance et al., 2010a, 2010b; Puente et al., 2011; Shah et al., 2009), the vast majority of which probably have no biological relevance. The accumulation of mutations in cancerous and precancerous cells over time is increasingly recognized as a complex, dynamic process. Carcinogenic exposures and DNA repair defects can lead to sustained elevations in mutation rate; telomere attrition and chromothripsis can drive massive genomic rearrangement in catastrophic bursts (Bignell et al., 2007; Campbell et al., 2010; O'Hagan et al., 2002; Stephens et al., 2011).

In the classic view of cancer development, those somatic mutations conferring a selective advantage on the cell drive successive waves of clonal expansion, with the fittest clone coming to dominate the cellular compartment. Increasingly, however, cancers are recognized to be mixtures of competing subclones, based on analyses of cancers sampled within a patient at different times (Ding et al., 2012; Shah et al., 2009), from different sites (Campbell et al., 2010; Ding et al., 2010; Yachida et al., 2010), at hypermutable genomic loci (Campbell et al., 2008), or through single-cell isolation (Anderson et al., 2011; Navin et al., 2011; Notta et al., 2011). Although these studies imply the existence of genetic heterogeneity within a tumor, fundamental questions remain about the dynamics of Darwinian evolution in cancer, the biological relevance of subclonal genetic variation and the relationship between mutational processes and clonal expansion.

Here, we use newly developed bioinformatic algorithms (Greenman et al., 2012) to reconstruct the genomic history of 21 breast cancers. Borrowing the concept of a "most-recent common ancestor" from population genetics, we can divide somatic mutations into those acquired before the last complete selective sweep (and thus shared by all cancer cells within the sample) and those subclonal variants that occurred after the emergence of the common ancestor. We study the early genomic evolution of the eventual cancer clone, quantify the extent and dynamics of subclonal variation within the cancer sample sequenced and explore changes in mutation signatures over time. These findings have important implications for our understanding of how breast cancers develop over the decades between breast organogenesis and diagnosis in the adult.

RESULTS

Inference of Cancer Genome Evolution

We sequenced 20 primary breast cancer samples to an average 30–40 coverage across each base in the genome. The sample series includes four cases each of estrogen-receptor (ER)-positive, *HER2*-positive, and *BRCA2*-positive breast cancer; three cases of triple negative; and five cases of *BRCA1*-positive breast cancer. In addition, we sequenced to 188-fold depth one other ER-positive tumor with a distinctive mutator phenotype, consisting of C>A, C>G and C>T mutations specifically in a TpC context. As described in the companion paper to this (Nik-Zainal et al., 2012, this issue of *Cell*), we identified a high-confidence, validated set of base substitutions, insertions, and deletions (indels); genomic rearrangements; and copy number changes in the 21 cancers.

To develop the reasoning that underpins this paper, we start with the tumor sequenced to 188-fold depth, PD4120a. At the chromosomal scale, the cancer genome is hypodiploid, with relatively few copy number changes (Figure 1A). To exploit the considerable sequencing depth available for this tumor, we modified the parameters of our somatic substitution algorithm in order to identify subclonal mutations; those found in only a fraction of tumor cells. In total, we identified 70,690 somatic substitutions genome-wide, including many in which fewer than 5% of the reads across the base reported the variant allele. That these are bona fide mutations is evidenced by the dominance of the C>* mutations in a TpC context at all levels of subclonality (Figures S1A and S1B, available online) and a high rate of verification in a subset by targeted PCR and pyrosequencing.

The mutations fall into well-circumscribed clusters when displayed by the fraction of reads reporting the variant (Figure 1B and Figure S1C). The major cluster of points occurs at a read depth around 210, with about 35% of reads reporting each variant. Because an estimated 70% of cells in the sample derive from the cancer clone, this cluster represents those point mutations found in all tumor cells on one copy of a diploid chromosome. By comparison, mutations from regions of copy number 1 show a lower overall read depth (because the copy number is lower) but higher variant allele fraction (because there are no reads from the deleted chromosome).

The genome has one triploid chromosomal region, 1q, which is most likely to have arisen as a single gain of one whole chromosome arm, although we cannot formally exclude duplication of both alleles with subsequent loss of one. Mutations occurring on the relevant chromosomal arm before duplication would be present on two of three copies (with an expected variant allele fraction of $\sim$55%), whereas mutations occurring after the duplication would be present on only one copy. In fact, we find only seven mutations on 1q predicted to have occurred before the chromosome arm was duplicated, not one of which has the signature of C>* mutations in a TpC context (Figure S1C). In contrast, 1,250 mutations are found on 1q at single copy number, of which 1,130 have this mutation signature, with the spectrum of early (ploidy 2) and late (ploidy 1) mutations being significantly different ($p < 0.0001$, chi-square test). Trisomy 1q is one of the commonest copy number alterations seen in breast cancer (Beroukhim et al., 2010; Bignell et al., 2010). Our data indicate that, relative to point mutations, this driver cytogenetic change occurred very early during the evolution of this particular tumor. Furthermore, albeit with small numbers of informative early mutations, the mutator phenotype was not evident before the occurrence of trisomy 1q.

Modeling Clonal and Subclonal Mutation Clusters

Many of the mutations we identify are present at a lower proportion of reads than we would expect for the ploidy and level of normal cell contamination in the sample (Figure 1B). These are subclonal mutations, found in only a fraction of the tumor cells. A particularly striking feature of these mutations is that they seemingly fall into distinct clusters.

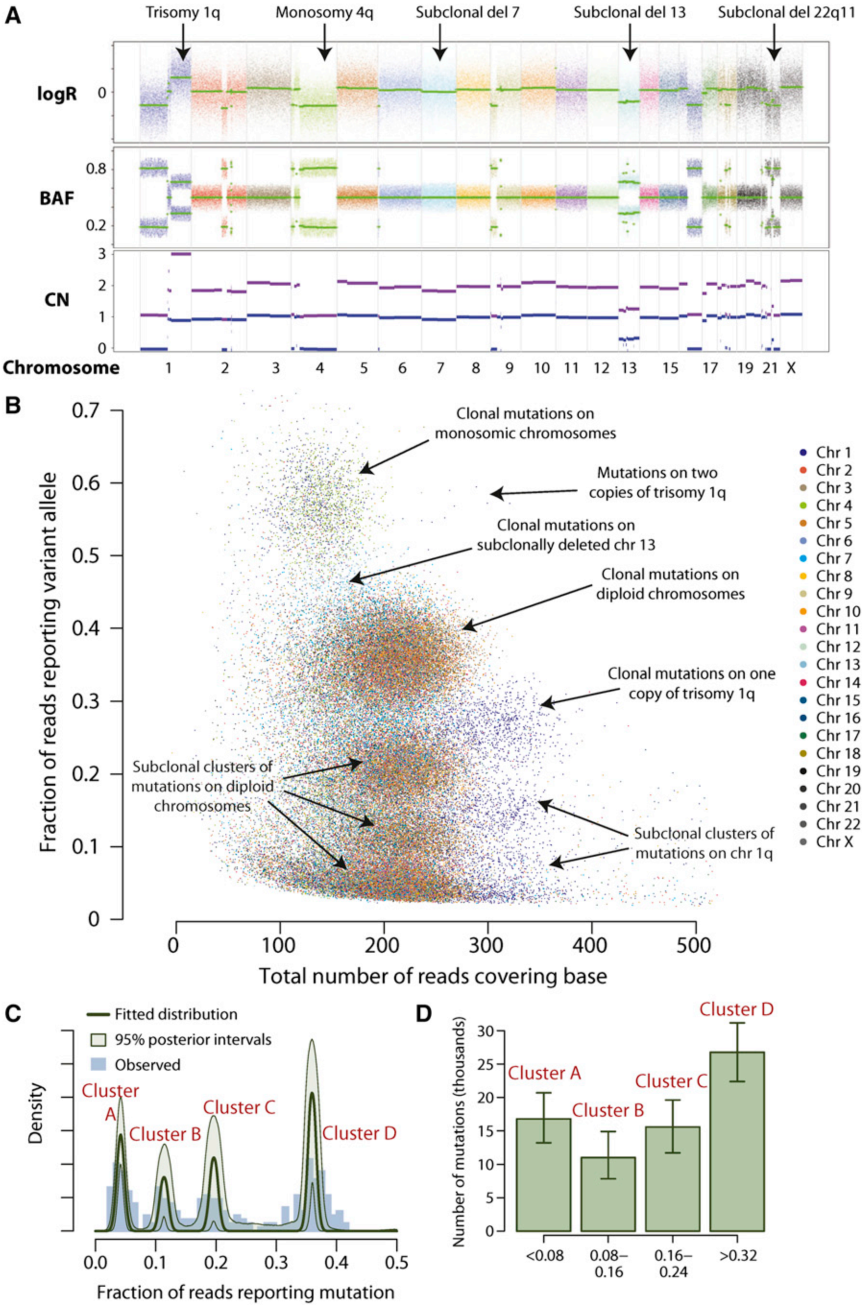

Figure 1. Genomic Architecture of PD4120a, a Breast Cancer Genome Sequenced to 188-Fold Coverage

(A) Copy number profile of the sample, with the upper panel showing the logR of intensity and the middle panel showing the B allele fraction (BAF) of germline heterozygous SNPs. Genomic segments of constant logR and BAF value were identified by the ASCAT algorithm (green lines). These were interpreted to give estimated overall copy number (purple lines) and copy number of the minor allele (blue lines) across the genome (lower panel).

(B) Distribution of 70,690 somatically acquired base substitutions according to the total number of reads across that base (x axis) and the fraction of those reads reporting the variant (y axis). Points are colored according to the chromosome the mutation derives from.

(C) Statistical modeling of the distribution of clonal and subclonal mutations by a Bayesian Dirichlet process. The empiric histogram of mutations is shown in pale blue, with the fitted distribution as a dark green line. Also shown are the 95% posterior confidence intervals for the fitted distribution (pale green area). Four separate clusters of mutations, named A–D, are identified.

(D) Estimated number of mutations found in clusters A–D, with the error bars representing the 95% posterior confidence intervals.

These data imply that the population of tumor cells within this breast cancer sample contains several discrete subclones, each of which represents a certain fraction of tumor cells and contains a certain number of substitutions (namely the size of the cluster). To enable formal development of this concept, we explicitly modeled the observed patterns of clonal and subclonal mutations with a hierarchical Bayesian Dirichlet process (Dunson, 2010). Using this flexible approach, we model the mutations as deriving from an unknown number of subclones, each of which is present at an unknown fraction of tumor cells and contributes an unknown proportion of all somatic mutations, with all the unknown parameters to be jointly estimated in the model (Extended Experimental Procedures).

The model performs well on simulated data sets, recapturing the "true" underlying distribution of subclones accurately, across variable numbers and sizes of subclonal populations (Figures S2A–S2C). We therefore applied this model to mutations found in PD4120a (Figure 1C and Figure S2D). This clearly shows four distinct clusters of mutations, one set found in all tumor cells (which we call cluster D henceforth) and three clusters of subclonal mutations, centered on variant allele fractions of 5% (cluster A), 11% (cluster B), and 19% (cluster C). From the model, we can generate estimates of the number of mutations found in each of these clusters together with 95% posterior confidence intervals for the estimates (Figure 1D).

The model predicts that some 26,762 mutations (95% posterior interval, 22,378–31,160) are found in all tumor cells in PD4120a. The implication is that during the evolution of this cancer, there was some ancestral cell that carried this complement of somatic mutations. Borrowing the term from population genetics, we term this cell the "most-recent common ancestor" of the tumor, and its emergence demarcates the split between mutations that are fully clonal and those that are subclonal. Among the mutations acquired before the emergence of the

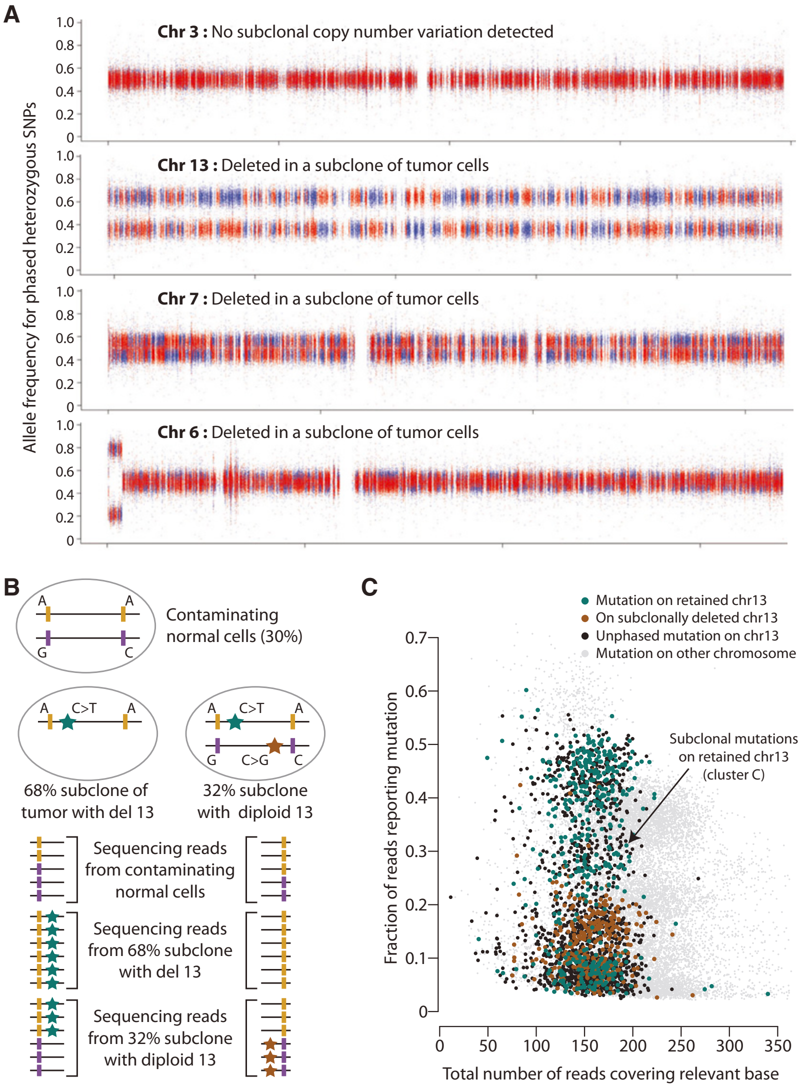

Figure 2. Subclonal Genetic Variation in PD4120a

(A) Battenberg plots of allele fractions for phased parental haplotypes for four chromosomes. Germline SNPs are phased by imputation, with observed allele fraction for one phased chromosomal copy plotted in blue and the other in red.

(B) Phasing of mutations (stars) with adjacent germline heterozygous SNPs (vertical lines) allows determination of whether a mutation is on the retained or subclonally deleted parental copy of a chromosome.

(C) Distribution of somatically acquired base substitutions on chromosome 13 according to the total number of reads across that base (x axis) and the fraction of those reads reporting the variant (y axis). Points are colored according to whether the mutation derives from the retained copy of chromosome 13 (green points), the subclonally deleted copy of chromosome 13 (brown points) or whether it could not be phased with a nearby heterozygous SNP (black points).

most-recent common ancestor are several in cancer genes, including *TP53*, *PIK3CA*, *GATA3*, *MLL3*, *SMAD4*, and *NCOR1*. In addition, the trisomy 1q described above had occurred, as well as an unbalanced t(1;22) translocation and a cluster of chromothripsis rearrangements involving chromosomes 2, 4, 18, and 21 (Figure S2E).

Subclonal Loss of Multiple Chromosomes in PD4120a

The copy number profile for chromosome 13 in PD4120a reveals that it is deleted in some, but not all, tumor cells (Figure 1A). The logR values, which measure total copy number, show decreased intensity compared to diploid chromosomes but higher than monosomic chromosomes. The allele fraction plot, which reports the relative proportions of the two alleles for heterozygous SNPs, shows similarly intermediate levels for chromosome 13. From these variables, we estimate that 68% of tumor cells have one copy of chromosome 13 deleted (95% confidence interval, 67%–69%). The same pattern is seen for 22q, indicating that the t(1;22) derivative chromosome has been deleted in a similar fraction of cells.

Allele frequency plots for chromosome 7 also appear slightly more widely distributed around 0.5 than other diploid chromosomes, associated with a concomitant small decrease in logR levels overall for this chromosome (Figure 1A), suggesting that the chromosome is lost in a minor fraction of tumor cells. With data from the 1000 Genomes Project (1000 Genomes Project Consortium, 2010), it is now possible to impute linkage of many germline SNPs into parent-specific haplotype blocks. We hypothesized that analysis of allele ratios by haplotype rather than individual SNP would draw out subtle deviations from the expected fraction of 0.5 with substantially improved statistical power and developed a bioinformatics method to assess this (the "Battenberg" algorithm; Extended Experimental Procedures). Applying it to chromosome 3 demonstrates no evidence for subclonal variation in copy number, as parent-specific allelic fractions in red and blue are superimposed at 0.5, as expected (Figure 2A). In contrast, for chromosome 7, red and blue patches marking parent-specific haplotypes show clear separation, indicative of subclonal deletion of the chromosome in a small fraction of tumor cells. Remarkably, when we apply this analysis genome-wide, we find that 14 chromosomes overall show statistically significant evidence for subclonal copy number variation (Figure 2A and Figure S3A). For these other regions, which include chromosomes 6, 8, 9, 11, 12, 14, and 15, the extent of separation is similar and less than that observed for chromosome 7.

Chromosome 2 shows an interesting pattern of changes. The logR values for "diploid" regions of chromosome 2, measuring overall copy number, are clearly lower than those for chromosome 5 (average logR, 0.020 versus 0.161; $p < 10^{-308}$) but virtually the same as for chromosome 7 (average logR, 0.020 versus 0.017; Figure S3B), implying that chromosome 2 is subclonally

deleted in a similar proportion of cells to chromosome 7. However, the haplotype-specific phasing analysis described above confirms that the allele fraction at "diploid" regions of the chromosome is exactly balanced at 0.5 (Figures S3A and S3B). The implication therefore is that chromosome 2 is subclonally deleted in PD4120a, but both parental copies have been lost in an exactly balanced proportion of cells.

In summary, these analyses indicate subclonal deletion of chromosome 13 in ~68% of tumor cells. There is also evidence for loss of chromosome 7 in a smaller fraction of cells, and for losses of other chromosomes, including 6, 8, 9, 11, 12, 14, 15, 18, and 21 in an even smaller proportion. Finally, there is convincing evidence that both parental copies of chromosome 2 have been lost in exactly equal proportions.

Integrating Subclonal Point Mutations and Copy Number Changes

As discussed above, there is a cluster of ~15,600 subclonal point mutations found at a variant allele fraction of ~19% (cluster C). Because this variant allele fraction is more than half that of the fully clonal mutations, each of these mutations is present in more than 50% of tumor cells. Consider any two of these mutations. By the so-called "pigeonhole principle," there must be at least one tumor cell that contains both mutations, because there is no way to apportion two lots of > 50% to completely separate subsets. Therefore, the two variants must be collinear on the phylogenetic tree. If this reasoning applies for any two such mutations at > 50%, then it applies to all such mutations en bloc. Furthermore, if one such mutation is found in a strictly greater fraction of cancer cells than another such mutation, then it must have occurred earlier than the other. Applying these deductions to PD4120a, it follows that the mutations found in cluster C are all on the same branch of the phylogenetic tree, together with the subclonal deletion of chromosome 13. Furthermore, because the deletion is found in a larger proportion of cells, it must have occurred earlier during the cancer's evolution than cluster C mutations.

We can directly test the veracity of this reasoning. Because we reason that the deletion of 13 occurred before subclonal mutations in cluster C, we predict that those subclonal mutations could only involve the retained copy of chromosome 13. Many somatic mutations will be sufficiently close to heterozygous germline SNPs that individual sequencing read pairs will span both, thus allowing the mutation to be "phased" with the SNP (Figure 2B). Of the 2,171 mutations on chromosome 13, we were able to phase 756 (35%) with a nearby heterozygous SNP, thus unambiguously determining whether the mutation occurred on the parental copy of chromosome 13 that was subclonally deleted or on the retained copy (Figure 2C). We find a cluster of mutations on the retained copy of chromosome 13 at a variant allele frequency of ~48% (green points, Figure 2C)—this represents fully clonal mutations (cluster D). We also see a cluster of mutations from the deleted copy of chromosome 13 at ~15% (brown points), denoting ancestral mutations subsequently deleted in 68% of tumor cells. A third distinct cluster of mutations is evident at ~25% of reads, the equivalent of cluster C. All of these, as predicted, are phased with the retained copy of chromosome 13.

This approach is also informative for the other subclonally deleted chromosomes. For mutations on the retained copy of chromosomes 6, 7, 8, and 11, we find clusters A–D as for non-subclonal diploid chromosomes (Figure 3A and data not shown). For mutations on the parental copies of chromosomes 6 and 7 that are subclonally deleted, cluster B is completely lost, whereas the others remain unchanged. This demonstrates that virtually all mutations in cluster B are on a separate phylogenetic branch from mutations in cluster C. Furthermore, the subclonal deletion of chromosome 6 and 7 must be collinear with the mutations in cluster B. The same patterns and reasoning apply to chromosome 8, 11, 12, 14, 15, 18 and 21. For chromosome 2, we find that cluster B is abolished on both parental copies of the chromosome. This confirms the observation from the logR values that both copies of chromosome 2 are subclonally deleted (Figure S2B) and moreover places these deletions on the same branch as the mutations in cluster B.

In summary, these data indicate that the subclonal deletion of chromosome 13 and the mutations in cluster C are on the same branch of the phylogenetic tree, with del13 occurring first. On a separate branch of the tree from this dominant subclone, we find all the mutations in cluster B, together with subsequent subclonal deletion of chromosomes 2, 6, 7, 8, 9, 11, 12, 14, 15, 18, and 21.

Phasing Pairs of Subclonal Somatic Mutations

We can also attempt to phase any two somatic mutations that are sufficiently close together to be spanned by single read pairs. We recognize two informative scenarios. The two mutations could arise in completely independent subclones, in which case reads could report either variant alone but never the two together (Figure S4A). Alternatively, one mutation could occur as subclonal evolution in a cell that already contains the other mutation, in which case we would see reads that report the earlier variant only as well as reads that report both variants together (Figure S4B). It is only valid to identify mutually exclusive mutation pairs in chromosomes that are haploid in the tumor, and we do indeed find 17 such pairs (Figure 3B and Figure S4C). Genome-wide, we also identify 76 examples of sub-subclonal evolution occurring on the same allele as a pre-existent subclonal mutation (Figure 3C, and Figure S4D). Strikingly, there are no examples of sub-subclonal evolution at 9%–12% variant allele fraction (cluster B) occurring in conjunction with a mutation at > 16% allele fraction (cluster C), confirming that mutations in cluster B fall on a separate phylogenetic branch from those in cluster C.

These data also indicate that cluster A, the set of mutations at a variant allele fraction ~5%, is likely to contain several discrete subclones. Some of these variants are clearly subclonal to cluster C and others subclonal to cluster B, as shown in Figure 3C. However, most are not derived from cluster B, because the peak for cluster A is largely unchanged for the parental copy of chromosome 7 that is subclonally deleted (Figure 3A). Mutations in cluster A frequently fell in mutually exclusive subclones (Figure 3B) and hence on different branches of the phylogenetic tree. It is therefore probable that some or even most of the cluster A mutations represent a third branch from the most-recent common ancestor. In support of this is a pair of cluster A

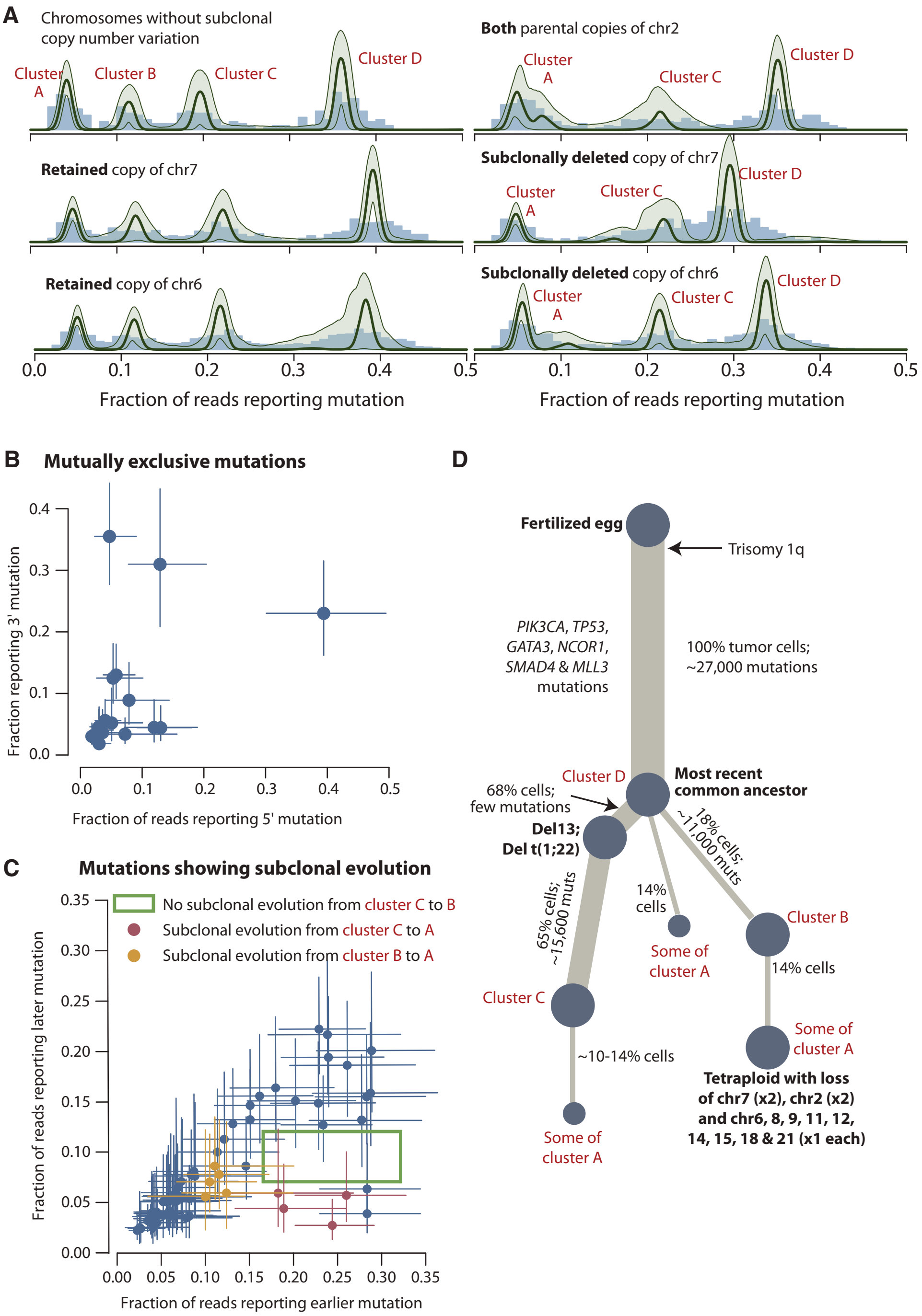

Figure 3. Reconstructing the Evolution of PD4120a

(A) Distribution of clonal and subclonal mutations phased onto specific chromosomes. The empiric histogram of mutations is shown in pale blue, with the fitted distribution and posterior intervals as dark green lines.

(B) Allele fractions for pairs of subclonal mutations that are found on separate branches of the phylogenetic tree, by virtue of no sequencing read evincing both mutations together. Error bars represent the 95% confidence intervals for the observed fractions.

(C) Allele fractions for pairs of subclonal mutations found in the same subclone, where one occurred temporally later than the other. Error bars represent the 95% confidence intervals for the observed fractions.

(D) Reconstruction of the phylogenetic tree for PD4120a. The thickness of the branches reflects the proportion of tumor cells comprising that lineage. The length of the branches reflects the number of mutations specific to that lineage.

mutations, both of which phase with the subclonally deleted copy of chromosome 13, and hence cannot be placed on the del13 branch, but are mutually exclusive with one another (Figure S4E).

A Phylogenetic Tree for PD4120a

With these observations, we can integrate the subclonal chromosome-scale losses with the subclonal point mutations to reconstruct how the tumor has evolved (Figure 3D). In one branch of the phylogenetic tree, there has been loss of chromosome 13 and subsequent acquisition of cluster C mutations. The other branch of the phylogenetic tree contains the cluster B mutations and sub-subclonal losses of multiple chromosomes, including both parental copies of chromosome 2. Because homozygous deletion of chromosome 2 in a diploid cell is frankly implausible, the most likely model is that a sub-subclone of the cluster B subclone has become tetraploid, presumably through an endoreduplication event. It has subsequently lost one of the four copies of chromosomes 6, 8, 9, 11, 12, 14, 15, 18, and 21. In addition, both copies of the same parental chromosome 7 have been lost, and one of each parental copy of chromosome 2 has been lost. From this model, we estimate that the subclone with mutations in cluster C represent 65% of tumor cells, cluster B represents 18% of tumor cells, and the tetraploid subclone represents 14% of tumor cells. Mutations in cluster A account for 14% of tumor cells. If many of these do fall on a third branch of the phylogenetic tree, the three branches would neatly account for all descendants of the most-recent common ancestor because the 14% of tumor cells in cluster A, the 68% with deletion 13, and the 18% of tumor cells in cluster B together add up to 100%. This phylogenetic tree explains all data observed for PD4120a.

Timing Chromosomal Evolution in 20 Breast Cancer Genomes

Chromosomal instability, the gains and losses of whole chromosomes or chromosome arms, is a well-recognized feature of breast cancer cells probably caused by missegregation of chromosomes during cell division (Burrell et al., 2010). As outlined above, for genomic regions that have increased in copy number, we can estimate the timing of the duplication event by comparing the proportion of mutations at ploidy 1 and ploidy 2 (Greenman et al., 2012). Among the 20 breast genomes sequenced to 30- to 40-fold depth, 16 had informative genomes for timing chromosomal gains (Figure 4). Broadly, the data suggest that the onset of large-scale chromosomal gains did not begin across these genomes until after at least 15%–20% of point mutation time had elapsed but thereafter continued steadily in many tumors. The implication is that chromosomal instability is not usually the earliest source of mutation in breast cancer evolution, but is a common and on-going process in later stages.

A related phenomenon is that of whole-genome duplication, caused by a single event of cytokinesis failure, endoreduplication, or fusion of two diploid cells. Ten of the tumors studied here show evidence for such an event, inferred from the homogeneity of the distribution of early and late mutations across the genome (Figure 4 and Figure S5A). In general, such endoreduplication was a late event in this series, occurring after more than 50% of point mutation time had elapsed and often following many preceding single chromosome losses and gains.

Five informative tumors had genomic amplifications of known cancer genes, three involving *ERBB2* and one each involving *MYC* and *CCND1*. In four of the five cases, no mutations were present on all copies of the amplified segment, even allowing for the one patient where both parental copies of the locus contributed to the amplification (Figures S5B and S5C). Therefore, the first rearrangement driving the genomic amplification presumably occurred early in the evolution of these cancers. Interestingly, however, all amplifications showed multiple mutations at several discrete stages of ploidy intermediate between all copies and only one copy of the amplified region (Figures S5B and S5C). These mutations must have accumulated after the amplification had begun, because they do not involve all copies, but before the amplification was complete, because their ploidy is more than 1. Coupled with the fact that such mutations were observed at several discrete levels of ploidy, these data suggest that the genomic rearrangements driving the amplifications in these patients were acquired over a relatively protracted period of molecular time. This pattern is different to mechanisms of genomic amplification such as breakage-fusion-bridge or double minute chromosomes, where amplification can occur rapidly and even exponentially over a few cell cycles (Bignell et al., 2007; Campbell et al., 2010; Stephens et al., 2011).

Changing Spectrum of Mutations over Time

In addition to timing when chromosomal gains occur, we can compare the spectrum of point mutations acquired early, before the copy number gain (ploidy 2), and late, after the gain (ploidy 1) (Pleasance et al., 2010a). Fourteen of the genomes had sufficient numbers of early and late mutations to enable statistical comparison of the mutation spectrum over time (Figure 5A). Of these, 11 had statistically significant differences in spectrum between mutations acquired early and late, with many patients showing a strikingly different profile.

The most consistent pattern is that C>T transitions constitute a higher proportion of early mutations than of late mutations, with 10/14 genomes showing a statistically significant decrease in C>T ratios after chromosome gains. C>T transitions are frequently caused by spontaneous deamination of methylated cytosine to thymine. However, we find that, in general, the decreased proportion of C>T mutations over time applies equally to other contexts as to those at CpG dinucleotides (Figure S6).

In the companion paper to this one (Nik-Zainal et al., 2012), we show that many breast cancer genomes have distinctive mutation processes, from which a nonnegative matrix factorization algorithm identified five separate signatures. By classifying whether mutations were early clonal (ploidy > 1), late clonal (ploidy = 1) or subclonal (ploidy < 1) in regions of copy number gains, we could assess the relative contributions of these five processes at different times during a cancer's evolution. In 8 patients, sufficient numbers of mutations were present in such regions to generate a stable solution (Figure 5B). This confirms that C>T mutations at CpG dinucleotides, termed "signature A," contributes a large proportion of the early mutations in these cancers, and relatively few late in the evolution of

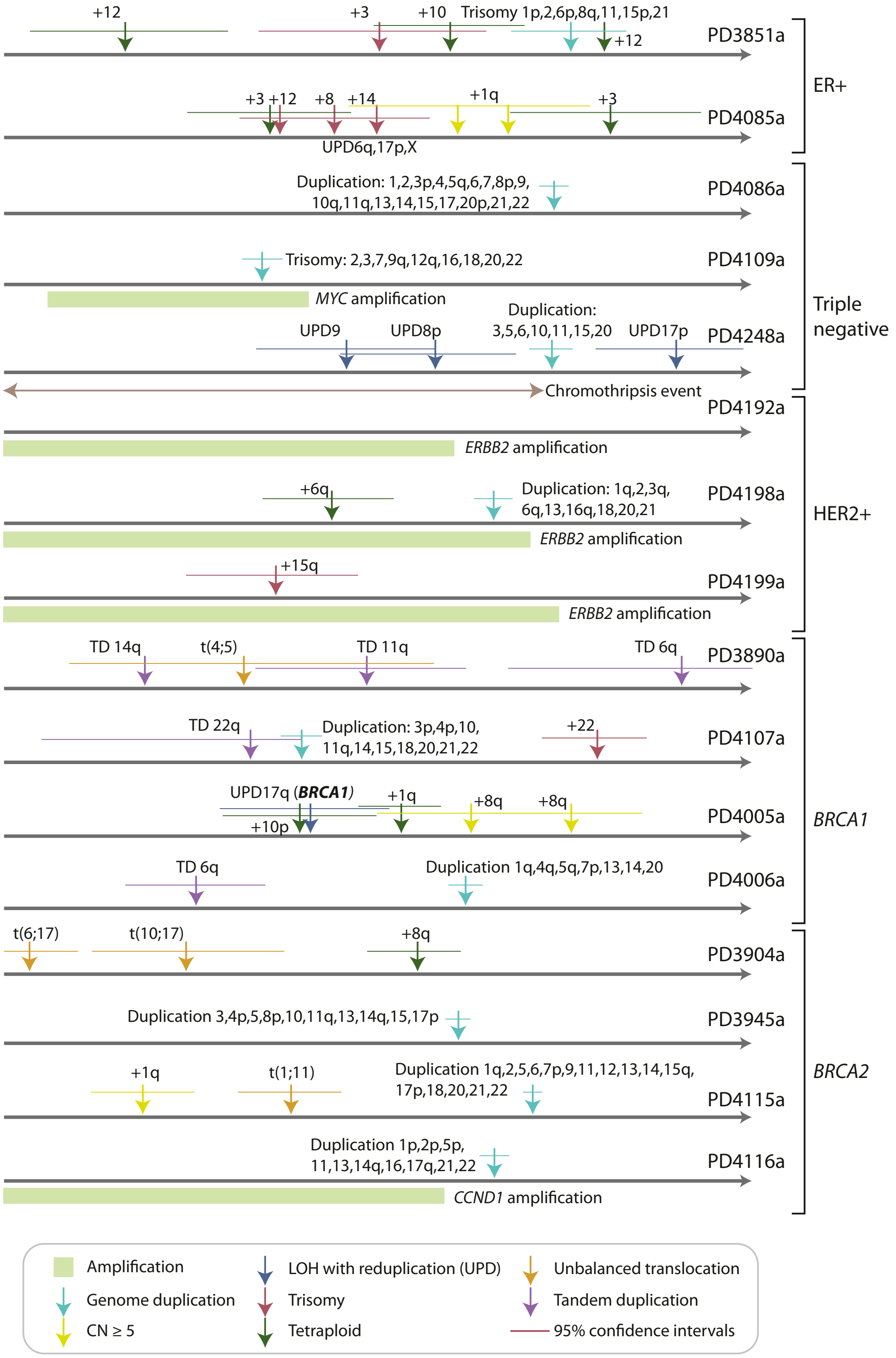

Figure 4. Timing of Copy Number Gains in 16 Informative Breast Cancer Genomes from the Ploidy of Mutations

The point estimates of timing for specific copy number gains are shown as arrows colored by the type of chromosomal aberration, with 95% confidence intervals generated by bootstrapping shown as horizontal lines. Molecular time is shown as an arrow, with the timing estimated as a fraction of point mutation time.

the tumors. In contrast, "signature E," denoting C>G mutations at Tp<u>C</u>pA, Tp<u>C</u>pC and Tp<u>C</u>pT trinucleotides, is a late onset mutational signature, contributing a large fraction of subclonal mutations in many patients. A similar pattern is seen for "signature B" comprising C>G or C>T mutations in a TpC context. Although not shown in these figures, dinucleotide substitutions

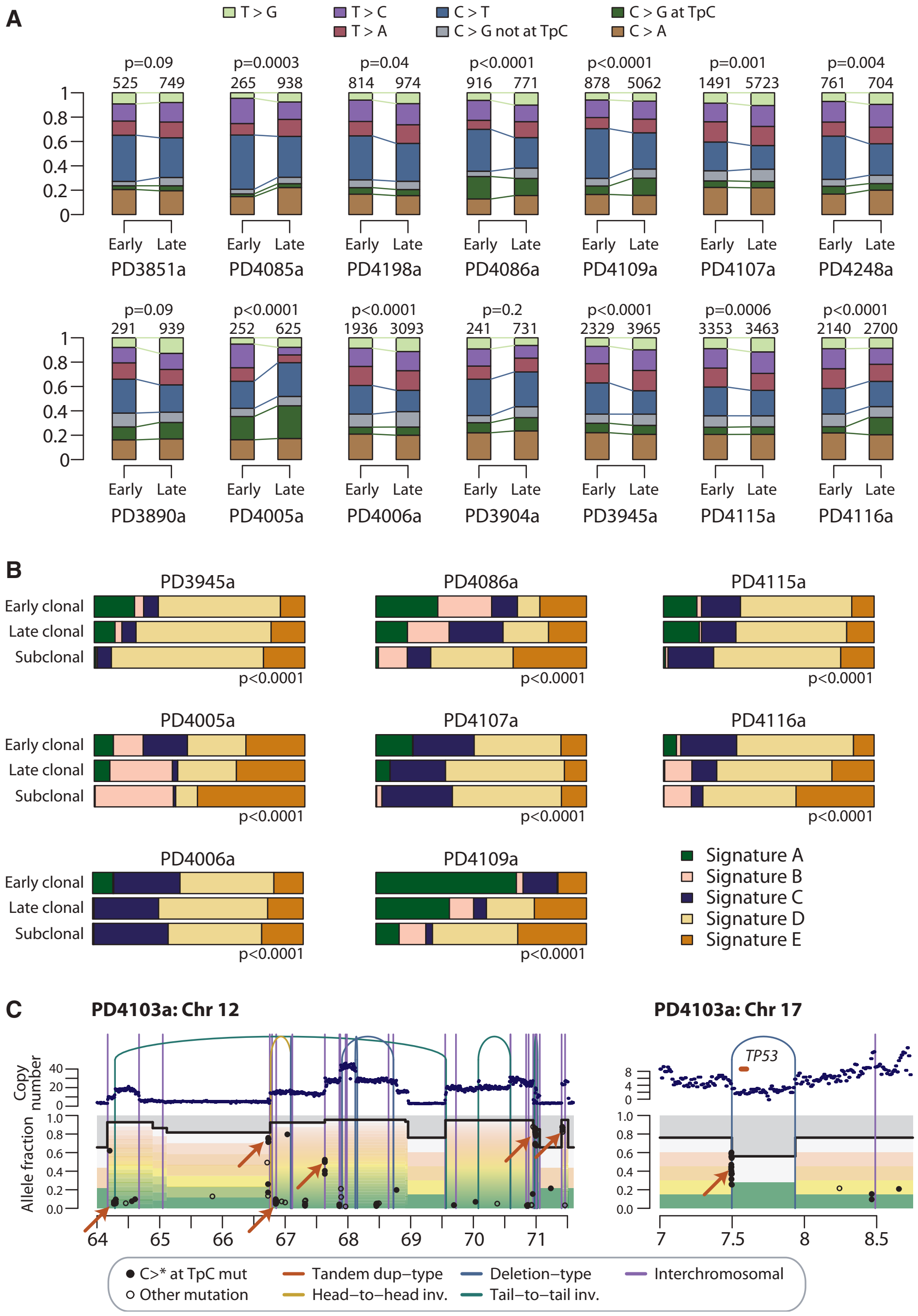

Figure 5. Comparison of Early and Late Point Mutation Signatures in 14 Informative Breast Cancer Genomes

(A) Stacked bar charts showing the fraction of early mutations (ploidy > 1) and late mutations (ploidy = 1) accounted for by each mutation type. The p values refer to the overall difference in distribution between early and late mutations (chi-square test). The numbers above each bar refer to the number of mutations in the early or late fraction.

(B) Stacked bar charts showing comparison of mutational processes identified by nonnegative matrix factorization. The comparison is across early clonal mutations (ploidy > 1), late fully clonal mutations (ploidy = 1) and subclonal mutations (ploidy < 1) for eight samples. Signature A describes C>T mutations at XpCpG trinucleotides. Signature B was composed predominantly of C>T, C>G mutations, and C>A mutations in a TpC context. Signature C and Signature D were relatively uniform processes across all 96 possible mutated trinucleotides. Signature E specifically identifies C>G mutations at TpCpA, TpCpC, and TpCpT trinucleotides.

(C) Timing of kataegis mutation clusters in PD4103a for the amplicon involving chromosome 12 (left) and a *TP53* deletion (right). The top panel shows the copy number profiles with genomic rearrangements. The lower panel shows the point mutations as filled black circles for C>* mutations in a TpC context (as for kataegis) and open circles for other types of mutation. The y axis denotes the variant allele fraction, divided by the colored bars into the proportions of reads derived from contaminating normal cells (gray bars) and the fraction coming from each copy of that segment in the tumor cells (multiple colored bars).

were also significantly over-represented among late mutations than early events (odds ratio, 1.9; $p < 0.0001$).

Taken together, these data indicate that the mutational forces fashioning the breast cancer genome vary over time. C>T transitions, both at CpG dinucleotides and in other sequence contexts, play a significant role in the early acquisition of mutations, accounting for up to 40% of mutations acquired before chromosomal gains. To some extent, the profile of base changes seen among many of the early breast cancer variants is a default mutation spectrum, closely mirroring that seen in tumor types such as blood, pancreatic, and brain cancers (Greenman et al., 2007; Jones et al., 2008; Papaemmanuil et al., 2011; Puente et al., 2011) and indeed in germline nucleotide substitutions (Hwang and Green, 2004). The lower proportional contribution of C>T transitions among late mutations is most likely caused by an increase in the rate of other mutation types because tumor-specific signatures account for much of the variation between early and late mutations. Intriguingly, we find that there are several mutation signatures at play in many of these patients, contributing varying proportions of mutations and with onset at different times during cancer evolution. The implication therefore is that in most breast cancers, the mutation rate increases in more advanced stages of tumor development, driven by distinctive, cancer-specific mutational processes. These processes continue past the emergence of the most-recent common ancestor, driving subclonal diversification within the tumor.

Timing Kataegis, Localized Clusters of Mutations

In the companion paper to this one (Nik-Zainal et al., 2012), we describe localized clusters of C>T and C>G mutations occurring in a TpC context closely associated with genomic rearrangements, which we termed *kataegis*. The presumption here is that an individual cluster of mutations occurs in a single event because of the close association with rearrangements and the fact that there is a strong strand bias within a cluster. Although the mutations within each cluster might occur simultaneously, however, the relative timing of different clusters of kataegis is not clear.

In PD4103a, there are many clusters of kataegis mutations genome-wide. Interestingly, within the amplicon involving regions of chromosomes 10, 11, and 12, we find that these clusters occur at several different levels of ploidy (Figure 5C). For example, on chromosome 12, there are several such events found at variant allele fraction of 0.8 or higher in association with rearrangements that demarcate large copy number changes. These must have occurred early in the genesis of the amplicon and then themselves been amplified by subsequent rearrangements. Interestingly, there is also a cluster at an allele fraction of 0.4 and several at allele fractions < 0.1. These must have occurred later in the genesis of the amplicon. In addition, rearrangements in PD4103a outside this amplicon are also associated with kataegis, such as a deletion of *TP53* (Figure 5C). The implication is that these clusters of mutations have not all occurred in a single event.

The other patient with particularly high numbers of these clusters, PD4107a, shows a somewhat different pattern. Here, the kataegis mutations are found specifically in association with a chromothripsis event on chromosome 6 and are all at the same level of ploidy. Thus, it seems very likely that both the chromothripsis and kataegis mutations did occur in the same catastrophic event. Nonetheless, elsewhere in this patient, there are other rearrangements with adjacent kataegis clusters, again arguing that this process can occur recurrently during the evolution of a breast cancer.

Dominant Subclones Are Always Present in Breast Cancers

In PD4120a, the high sequencing depth enables us to infer the existence of several subclonal expansions. For the 20 genomes sequenced at 30- to 40-fold coverage, there is a measurable probability that a sufficient number of reads will report a subclonal variant to allow our algorithm to call the mutation. We estimated this probability by using a statistical resampling method known as bootstrapping (Extended Experimental Procedures). On average for the 20 genomes, we have an approximately 90% chance of detecting a fully clonal mutation, a 60% chance of detecting a mutation found in 50% of tumor cells, and a 5% chance of detecting a mutation in 25% of tumor cells (Figure S7A). For 19 of the genomes reported here, 150–300 somatic substitutions were independently verified by PCR and deep pyrosequencing on the 454 platform, giving accurate estimates of the variant allele fraction for these mutations. For four samples in which exome pull-down and sequencing was also performed, the empiric distributions of subclonal mutations called in the original genome and subsequently validated by deep pyrosequencing or exome pull-down are very similar (Figure S7B).

We therefore applied the Bayesian Dirichlet process described earlier to the deep sequencing data, adding correction for the sample-specific sensitivity for detecting mutations at different levels of subclonality (Figures 6A and 6B and Figure S7C). We find subclonal point mutations in all samples studied and indeed the estimated number of these genome-wide is, for most samples studied, more than the number of fully clonal mutations (Figure 6A). As for PD4120a, we always find evidence for a dominant subclonal lineage, comprising mutations found in 50%–95% of tumor cells, which the pigeonhole principle dictates must all be on the same branch of the phylogenetic tree. The patterns and distribution of this subclonal variation show diversity across the genomes, with some samples composed of a broad range of subclones at differing fractions of tumor cells (PD4088a, PD3905a, and PD4199a) and others showing fewer, more distinct subclonal expansions (PD4116a, PD4005a, and PD4085a). Overall, however, by the arguments above, all show a single dominant lineage of subclonal and sub-subclonal expansions within the population of cancer cells.

We can also apply the "Battenberg" analysis used for PD4120a, in which we phase haplotypes of germline heterozygous SNPs, to investigate subclonal copy number gains and losses in the other 20 genomes (Figure 6C). Again, we find considerable diversity in the frequency and patterns of subclonal regional variation. Some samples, such as PD4088a and PD4248a, showed very little subclonal copy number variation, but in other genomes, such as PD3851a, PD4085a, and PD4116a, the vast majority of the genome varies in copy number

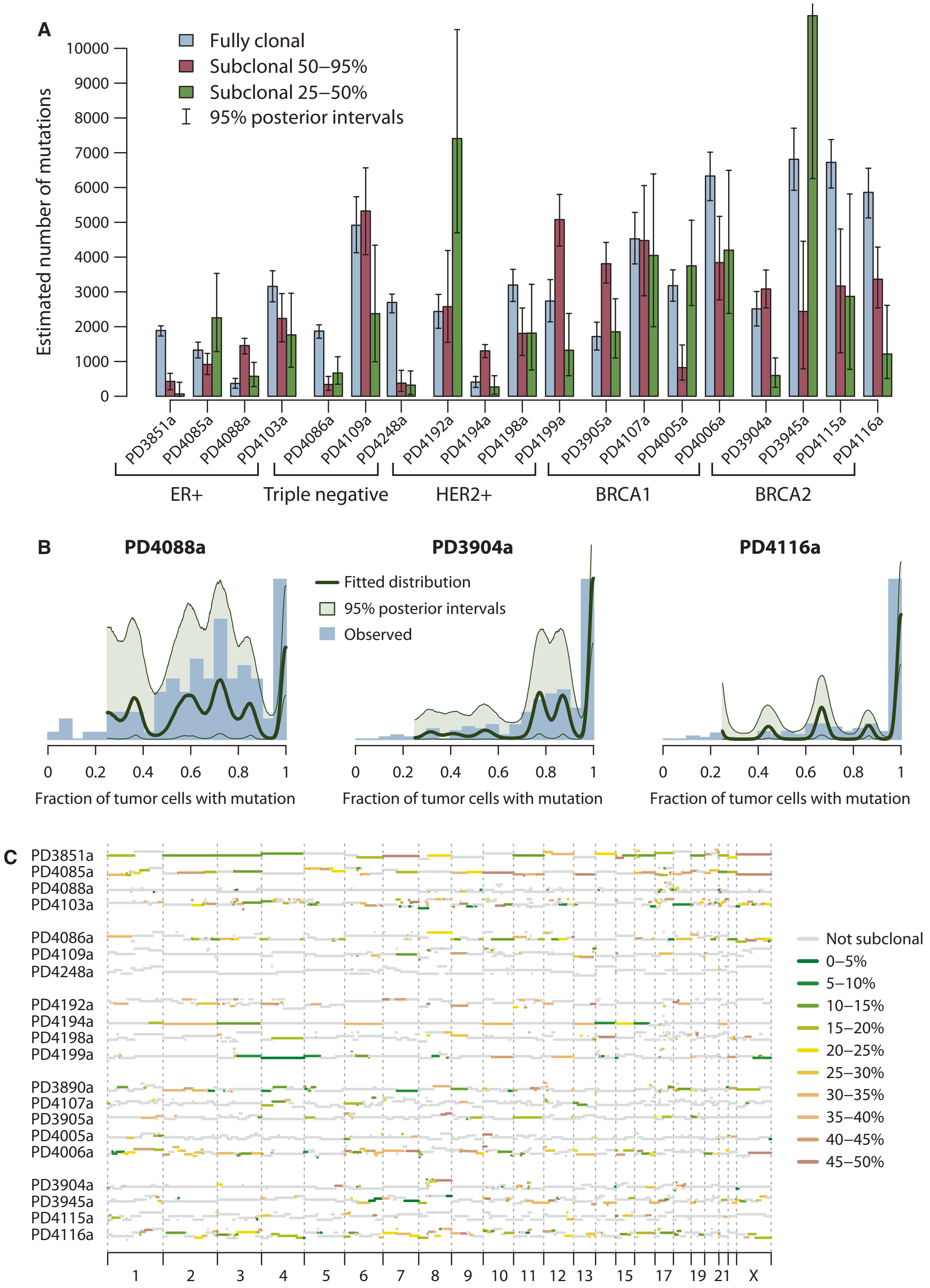

Figure 6. Subclonal Genetic Variation among 20 Breast Cancers

(A) Bar chart showing point estimates and 95% posterior confidence intervals for the number of fully clonal mutations (blue bars), mutations found in 50%–95% tumor cells (pink bars), and 25%–50% tumor cells (green bars).

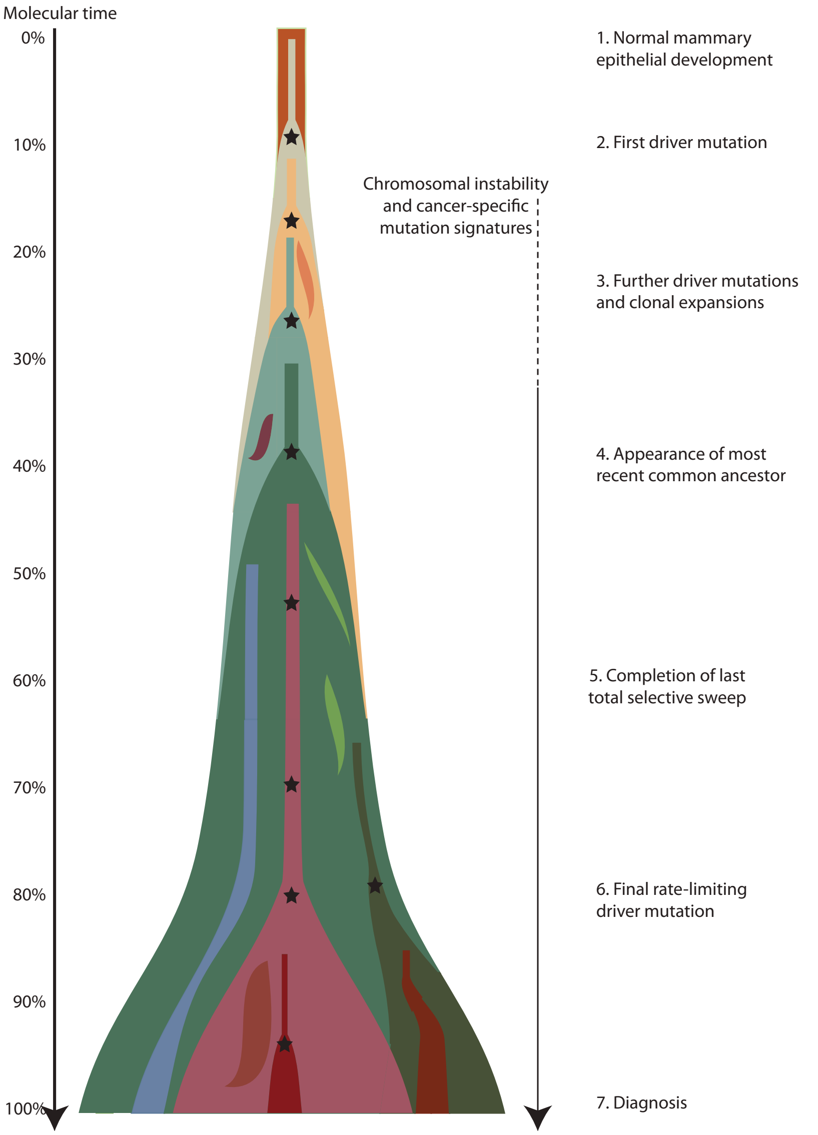

Figure 7. A Model for Breast Cancer Development over Molecular Time

The cancer evolves through acquisition of driver mutations (black stars), which produce clonal expansions. These driver mutations occur only infrequently in long-lived lineages of cells, which passively accumulate many mutations without expansion.

among different subclones within the cancer. Among these genomes, there is also diversity in the number of distinct subclones evident by this analysis. For example, PD4192a shows strong evidence on the Battenberg analysis for a subclone of 40%–50% of tumor cells with regional differences in copy number from other subclones across 10–12 chromosomes. Interestingly, this is matched by a discrete peak of point mutations in 40%–50% of tumor cells (Figure S7C). In contrast, many other genomes, such as PD4086a and PD3890a, show evidence for several distinct levels of subclonality across the genome. For three of these samples, we can apply similar reasoning as used for PD4120a to reconstruct the phylogenetic tree of the cancer (Figure S8).

In summary, these data indicate that a considerable proportion of somatic genetic variation in these 20 genomes is found in only a fraction of tumor cells. There is heterogeneity among different cases, but as a general rule, there is always a dominant subclonal lineage separated from the most-recent common ancestor by several hundreds to thousands of mutations.

DISCUSSION

A Model of Breast Cancer Development

From the analyses described here, we can begin to understand the dynamics of breast cancer development (Figure 7). A key landmark in this evolution is the appearance of the most-recent common ancestor—the cell that has the full complement of somatic mutations found in all tumor cells. All extant cancer cells in the sample analyzed can trace a genealogy back to the fertilized egg through this common ancestor, and its emergence demarcates the split in the phylogenetic tree from the shared trunk to the branches of divergent subclones. Our data consistently indicate that the most-recent common ancestor appeared surprisingly early in molecular time, or, expressed another way, much of molecular time is spent driving subclonal diversification and evolution among the nascent cancer cells. This is different to what is observed for acute myeloid leukemia, where the proportion of mutations that are subclonal is relatively small (Ding et al., 2012).

Before the appearance of the most-recent common ancestor, much oncogenic genetic change has accumulated in the lineage. Many of the tumors studied here have several driver mutations that are found in all tumor cells—all *PIK3CA* and *TP53* mutations, all *ERBB2*, *MYC*, and *CCND1* amplifications, all somatic loss of the wild-type *BRCA1* and *BRCA2* alleles among these 21 cancers can be placed unequivocally on the shared trunk of the phylogenetic tree. Chromosomal instability appears in

(B) Distribution of clonal and subclonal mutations for three representative cancers. The empiric histogram of mutations is shown in pale blue, with the fitted distribution and 95% posterior intervals as dark green lines.

(C) Subclonal copy number variation for the 20 breast cancer genomes, estimated by using the Battenberg algorithm. The height of each bar reflects the estimated copy number, and segments are colored by whether they show no subclonal variation (gray) or the estimated frequency of the minor subclone at the given region (green to yellow to brown).

many of the tumors from about 15%–20% of the way through molecular time and has an on-going impact thereafter, even beyond the appearance of the most-recent common ancestor. This results in the clonal acquisition of many recurrent abnormalities, such as gains of 1q and 8q and losses of 17p, and considerable divergence among subclones of the cancer in their large-scale chromosomal composition. Like chromosomal instability, other cancer-specific point mutational processes materialize during the tumor's development, having considerable impact on the number and patterns of late mutations.

Several profound insights into the patterns and dynamics of subclonal evolution, occurring after the appearance of the most-recent common ancestor, can be drawn from these 21 breast cancers. All of the tumors contained a dominant subclonal lineage, accounting for more than 50% of cancer cells in the sample and carrying many hundreds or thousands of point mutations. There is no a priori reason why a cancer should have such a dominant subclone, nor why the phylogenetic branch should carry so many mutations. The one unifying factor for all these tumors is, rather obviously, that they have been diagnosed: in other words, they are sufficiently large to be palpable or seen on a mammogram. In a breast cancer of typical size, 10 cm^3 say, the expansion of a subclone that ultimately constitutes 60% of tumor cells will contribute 6 cm^3 to the tumor bulk, assuming stromal contamination and cell size are proportionate. Such a significant fraction of a tumor's mass is likely to have a substantial impact on whether a lesion is clinically detectable or not.

The implication, therefore, is that expansion of a dominant subclonal lineage is the final rate-limiting step in the development of breast cancer, triggering diagnosis. Two important observations underpin this logic. First, the dominant subclone is separated from the most-recent common ancestor by many hundreds to thousands of point mutations, often more than the set of mutations shared by all cancer cells. Second, there is minimal evidence of significant clonal expansion before the accumulation of all mutations in the dominant subclone. This is particularly clearly demonstrated with the high sequence coverage for PD4120a. The dominant subclone here has some ~15,600 mutations found in 65% of tumor cells, with very few subclonal mutations found in more than 65% of cells. The event triggering the expansion of this subclone, presumably a somatic mutation, must therefore be rate limiting in the sense that Armitage and Doll (1954) use, because so many mutations stack up before the subclone begins proliferation.

Thus we glimpse a model of long-lived, but sparse, lineages of cells passively accumulating mutations until provoked into a major quest for tumor dominance. It is only when this subclone has grown sufficiently populous that the tumor mass becomes clinically detectable. For the tumors studied here, the number of mutations acquired after the split from the most-recent common ancestor in the lineage that becomes the dominant subclone is often similar to or more than the number acquired before the split, a striking finding given that several driver mutations are already present in the common ancestor. Our model has an obvious similitude with the concept of cancer stem cells—infrequent, self-renewing, metabolically quiescent cells capable of reconstituting a tumor (Anderson et al., 2011; Notta et al., 2011; Visvader and Lindeman, 2008).

The cancer genome is like a palimpsest, an ancient parchment that was frequently reused, each time retaining traces of what had previously been written. The interplay of point mutations, chromosomal gains and losses, and clonal expansions, acquired in a given temporal sequence, leave an analogous record of the life history of a cancer inscribed in its genome.

EXPERIMENTAL PROCEDURES

The protocols for sequencing and bioinformatics analysis for identification of somatic substitutions, indels, copy number changes and genomic rearrangements are all described in the companion paper to this one (Nik-Zainal et al., 2012). Estimates of normal cell contamination were derived by using the ASCAT algorithm, based on analysis of the B allele fraction for heterozygous germline SNPs for regions departing from diploidy in the tumor genome (Van Loo et al., 2010). For PD4120a, this estimate includes a correction for the fact that there is a tetraploid subclone.

For analyzing the subclonal structure of PD4120a and the other breast cancer genomes, we developed several new bioinformatics algorithms. These include methods for (1) phasing mutations with nearby heterozygous germline SNPs; (2) phasing pairs of subclonal mutations in close proximity; (3) identifying large-scale subclonal copy number variation (the Battenberg algorithm); and (4) modeling the clusters of subclonal base substitutions from deep coverage data by using Bayesian Dirichlet processes. These algorithms are described in step-by-step detail in Extended Experimental Procedures.

ACCESSION NUMBERS

The European Genome-Phenome Archive accession number for the sequence reported in this paper is EGAD00001000138. The ArrayExpress Archive accession number for the SNP6 array data reported in this paper is E-MTAB-1087.

SUPPLEMENTAL INFORMATION

Supplemental Information includes Extended Experimental Procedures and eight figures and can be found with this article online at doi:10.1016/j.cell.2012.04.023.

ACKNOWLEDGMENTS

This work was supported by the Wellcome Trust (grant reference 098051) and Breakthrough Breast Cancer Research (ICGC 08/09). S.N.-Z. is a Wellcome Trust Clinical Research Training Fellow. P.J.C. is a Wellcome Trust Senior Clinical Research Fellow (WT088340MA). P.V.L. is a postdoctoral researcher of the Research Foundation-Flanders. A.S. is supported by the HL Holmes Award from the National Research Council Canada and a Fellowship from EMBO. I.V. is supported by a fellowship from the International Human Frontier Science Program Organization. J.W.M.M. is funded in part by a research grant from the Netherlands Genomics Initiative (NGI)/Netherlands Organization for Scientific Research (NWO). A.-L.B.-D. and A.L. are funded by the Norwegian Research Council, Norwegian Cancer Society, Radium Hospital Foundation and Health Region South-East. S.M. is funded by the BASIS project through the European Community's FP7/2010-2014 under grant agreement 242006. We acknowledge the Core Sequencing Facility and IT groups of the Wellcome Trust Sanger Institute, support for samples, sample banking, and processing from the Breakthrough Breast Cancer Unit, members of the ICGC Breast Cancer Working Group, the Pathology Review subgroup of the Breast Cancer Working Group, the OSBREAC Consortium of The Oslo University Hospital and the National Cancer Institute Specialized Program Of Research Excellence (grant reference CA089393). We also acknowledge Dr. Peter Watson, the BCCA Tumour Tissue Repository, and the Centre for Translational Genomics. The authors acknowledge financial support from the Department of Health via the National Institute for Health Research comprehensive Biomedical Research Centre award to Guy's and St Thomas' NHS Foundation Trust.

Received: December 14, 2011
Revised: March 13, 2012
Accepted: April 29, 2012
Published online: May 17, 2012

REFERENCES

1000 Genomes Project Consortium. (2010). A map of human genome variation from population-scale sequencing. Nature *467*, 1061–1073.

Anderson, K., Lutz, C., van Delft, F.W., Bateman, C.M., Guo, Y., Colman, S.M., Kempski, H., Moorman, A.V., Titley, I., Swansbury, J., et al. (2011). Genetic variegation of clonal architecture and propagating cells in leukaemia. Nature *469*, 356–361.

Armitage, P., and Doll, R. (1954). The age distribution of cancer and a multi-stage theory of carcinogenesis. Br. J. Cancer *8*, 1–12.

Beroukhim, R., Mermel, C.H., Porter, D., Wei, G., Raychaudhuri, S., Donovan, J., Barretina, J., Boehm, J.S., Dobson, J., Urashima, M., et al. (2010). The landscape of somatic copy-number alteration across human cancers. Nature *463*, 899–905.

Bignell, G.R., Santarius, T., Pole, J.C., Butler, A.P., Perry, J., Pleasance, E., Greenman, C., Menzies, A., Taylor, S., Edkins, S., et al. (2007). Architectures of somatic genomic rearrangement in human cancer amplicons at sequence-level resolution. Genome Res. *17*, 1296–1303.

Bignell, G.R., Greenman, C.D., Davies, H., Butler, A.P., Edkins, S., Andrews, J.M., Buck, G., Chen, L., Beare, D., Latimer, C., et al. (2010). Signatures of mutation and selection in the cancer genome. Nature *463*, 893–898.

Burrell, R.A., Juul, N., Johnston, S.R., Reis-Filho, J.S., Szallasi, Z., and Swanton, C. (2010). Targeting chromosomal instability and tumour heterogeneity in HER2-positive breast cancer. J. Cell. Biochem. *111*, 782–790.

Campbell, P.J., Pleasance, E.D., Stephens, P.J., Dicks, E., Rance, R., Goodhead, I., Follows, G.A., Green, A.R., Futreal, P.A., and Stratton, M.R. (2008). Subclonal phylogenetic structures in cancer revealed by ultra-deep sequencing. Proc. Natl. Acad. Sci. USA *105*, 13081–13086.

Campbell, P.J., Yachida, S., Mudie, L.J., Stephens, P.J., Pleasance, E.D., Stebbings, L.A., Morsberger, L.A., Latimer, C., McLaren, S., Lin, M.L., et al. (2010). The patterns and dynamics of genomic instability in metastatic pancreatic cancer. Nature *467*, 1109–1113.

Chapman, M.A., Lawrence, M.S., Keats, J.J., Cibulskis, K., Sougnez, C., Schinzel, A.C., Harview, C.L., Brunet, J.P., Ahmann, G.J., Adli, M., et al. (2011). Initial genome sequencing and analysis of multiple myeloma. Nature *471*, 467–472.

Ding, L., Ellis, M.J., Li, S., Larson, D.E., Chen, K., Wallis, J.W., Harris, C.C., McLellan, M.D., Fulton, R.S., Fulton, L.L., et al. (2010). Genome remodelling in a basal-like breast cancer metastasis and xenograft. Nature *464*, 999–1005.

Ding, L., Ley, T.J., Larson, D.E., Miller, C.A., Koboldt, D.C., Welch, J.S., Ritchey, J.K., Young, M.A., Lamprecht, T., McLellan, M.D., et al. (2012). Clonal evolution in relapsed acute myeloid leukaemia revealed by whole-genome sequencing. Nature *481*, 506–510.

Dunson, D.B. (2010). Nonparametric Bayes applications to biostatistics. In Bayesian Nonparametrics, N.L. Hjort, C. Holmes, P. Müller, and S.G. Walker, eds. (Cambridge: Cambridge University Press).

Greenman, C., Stephens, P., Smith, R., Dalgliesh, G.L., Hunter, C., Bignell, G., Davies, H., Teague, J., Butler, A., Stevens, C., et al. (2007). Patterns of somatic mutation in human cancer genomes. Nature *446*, 153–158.

Greenman, C.D., Pleasance, E.D., Newman, S., Yang, F., Fu, B., Nik-Zainal, S., Jones, D., Lau, K.W., Carter, N., Edwards, P.A., et al. (2012). Estimation of rearrangement phylogeny for cancer genomes. Genome Res. *22*, 346–361.

Hanahan, D., and Weinberg, R.A. (2011). Hallmarks of cancer: the next generation. Cell *144*, 646–674.

Hornsby, C., Page, K.M., and Tomlinson, I.P. (2007). What can we learn from the population incidence of cancer? Armitage and Doll revisited. Lancet Oncol. *8*, 1030–1038.

Hwang, D.G., and Green, P. (2004). Bayesian Markov chain Monte Carlo sequence analysis reveals varying neutral substitution patterns in mammalian evolution. Proc. Natl. Acad. Sci. USA *101*, 13994–14001.

Jones, S., Zhang, X., Parsons, D.W., Lin, J.C., Leary, R.J., Angenendt, P., Mankoo, P., Carter, H., Kamiyama, H., Jimeno, A., et al. (2008). Core signaling pathways in human pancreatic cancers revealed by global genomic analyses. Science *321*, 1801–1806.

Ley, T.J., Ding, L., Walter, M.J., McLellan, M.D., Lamprecht, T., Larson, D.E., Kandoth, C., Payton, J.E., Baty, J., Welch, J., et al. (2010). DNMT3A mutations in acute myeloid leukemia. N. Engl. J. Med. *363*, 2424–2433.

Navin, N., Kendall, J., Troge, J., Andrews, P., Rodgers, L., McIndoo, J., Cook, K., Stepansky, A., Levy, D., Esposito, D., et al. (2011). Tumour evolution inferred by single-cell sequencing. Nature *472*, 90–94.

Nik-Zainal, S., Alexandrov, L.B., Wedge, D.C., Van Loo, P., Greenman, C.D., Raine, K., Jones, D., Hinton, J., Marshall, J., and Stebbings, L.A. (2012). Mutational Processes Molding the Genomes of 21 Breast Cancers. Cell *149*. Published online May 17, 2012. 10.1016/j.cell.2012.04.024.

Notta, F., Mullighan, C.G., Wang, J.C., Poeppl, A., Doulatov, S., Phillips, L.A., Ma, J., Minden, M.D., Downing, J.R., and Dick, J.E. (2011). Evolution of human BCR-ABL1 lymphoblastic leukaemia-initiating cells. Nature *469*, 362–367.

O’Hagan, R.C., Chang, S., Maser, R.S., Mohan, R., Artandi, S.E., Chin, L., and DePinho, R.A. (2002). Telomere dysfunction provokes regional amplification and deletion in cancer genomes. Cancer Cell *2*, 149–155.

Papaemmanuil, E., Cazzola, M., Boultwood, J., Malcovati, L., Vyas, P., Bowen, D., Pellagatti, A., Wainscoat, J.S., Hellstrom-Lindberg, E., Gambacorti-Passerini, C., et al; Chronic Myeloid Disorders Working Group of the International Cancer Genome Consortium. (2011). Somatic SF3B1 mutation in myelodysplasia with ring sideroblasts. N. Engl. J. Med. *365*, 1384–1395.

Pleasance, E.D., Cheetham, R.K., Stephens, P.J., McBride, D.J., Humphray, S.J., Greenman, C.D., Varela, I., Lin, M.L., Ordóñez, G.R., Bignell, G.R., et al. (2010a). A comprehensive catalogue of somatic mutations from a human cancer genome. Nature *463*, 191–196.

Pleasance, E.D., Stephens, P.J., O’Meara, S., McBride, D.J., Meynert, A., Jones, D., Lin, M.L., Beare, D., Lau, K.W., Greenman, C., et al. (2010b). A small-cell lung cancer genome with complex signatures of tobacco exposure. Nature *463*, 184–190.

Puente, X.S., Pinyol, M., Quesada, V., Conde, L., Ordóñez, G.R., Villamor, N., Escaramis, G., Jares, P., Beà, S., González-Díaz, M., et al. (2011). Whole-genome sequencing identifies recurrent mutations in chronic lymphocytic leukaemia. Nature *475*, 101–105.

Shah, S.P., Morin, R.D., Khattra, J., Prentice, L., Pugh, T., Burleigh, A., Delaney, A., Gelmon, K., Guliany, R., Senz, J., et al. (2009). Mutational evolution in a lobular breast tumour profiled at single nucleotide resolution. Nature *461*, 809–813.

Stephens, P.J., Greenman, C.D., Fu, B., Yang, F., Bignell, G.R., Mudie, L.J., Pleasance, E.D., Lau, K.W., Beare, D., Stebbings, L.A., et al. (2011). Massive genomic rearrangement acquired in a single catastrophic event during cancer development. Cell *144*, 27–40.

Stratton, M.R., Campbell, P.J., and Futreal, P.A. (2009). The cancer genome. Nature *458*, 719–724.

Van Loo, P., Nordgard, S.H., Lingjærde, O.C., Russnes, H.G., Rye, I.H., Sun, W., Weigman, V.J., Marynen, P., Zetterberg, A., Naume, B., et al. (2010). Allele-specific copy number analysis of tumors. Proc. Natl. Acad. Sci. USA *107*, 16910–16915.

Visvader, J.E., and Lindeman, G.J. (2008). Cancer stem cells in solid tumours: accumulating evidence and unresolved questions. Nat. Rev. Cancer *8*, 755–768.

Yachida, S., Jones, S., Bozic, I., Antal, T., Leary, R., Fu, B., Kamiyama, M., Hruban, R.H., Eshleman, J.R., Nowak, M.A., et al. (2010). Distant metastasis occurs late during the genetic evolution of pancreatic cancer. Nature *467*, 1114–1117.

EXTENDED EXPERIMENTAL PROCEDURES

Reconstructing the Evolution of Tumors

To reconstruct the genomic evolution of the 21 breast cancer genomes, we followed the approach we have described previously (Greenman et al., 2012). In particular, timing the onset of chromosomal gains from the proportions of mutations at ploidy 1 versus ploidy 2 followed the process outlined there. To estimate the 95% confidence intervals around the point estimates of timing, we bootstrapped the mutations identified in the relevant chromosomal region. For example, suppose there were 100 mutations at ploidy 1 and 20 mutations at ploidy 2 on a chromosome that is triploid with two copies of one parental chromosome and one copy of the other. Then the point estimate of when the extra copy of the chromosome was gained is 43% of molecular time (= 20/(20 + (100 − 20)/3)). We generate 10,000 bootstrap samples by resampling 120 mutations with replacement from the original 120. From the recalculated timing for each bootstrap sample, we can estimate the 95% confidence interval for the original point estimate.

To compare the distribution of mutation signatures between early and late mutations, we only use genomic regions of uniparental disomy, triploidy, and tetraploidy. Then, across such regions of the genome, all mutations at ploidy > 1 are aggregated as "early," and all ploidy 1 mutations are aggregated as "late." We compare the proportions of each by using standard chi-square tests for independence. Note that this introduces two simplifications. First, not all chromosomal gains occurred at the same time in these tumors, meaning that aggregation across different regions somewhat dilutes the differences observed. Second, in regions of triploidy as 2+1 parental copies, early mutations on the nonamplified parental copy will remain at ploidy 1 and thus be classified as "late." However, the effects of both of these simplifications would be to obscure any true differences between early and late mutation signatures, rather than make any artifactually appear. In practice, the effects of the simplifications are rather small, and outweighed by the improvements gained in ease of interpretation. We also applied the nonnegative matrix factorization, as described in the companion paper, to these timed mutations, including a category for subclonal mutations, where the variant allele fraction was < 75% of that expected for a fully clonal mutation at ploidy 1.

Mutation Analysis in PD4120a

To facilitate the identification of subclonal mutations in PD4120a, for which the genome was sequenced to an average 188-fold depth, we ran our substitution-calling algorithm by using a copy number of 10 throughout the tumor genome. Because the cancer genome in this sample is actually hypodiploid, this effectively allows the identification of mutations found in only a fraction of the tumor cells. The same post-processing filters as applied to cancer genomes sequenced with 30-fold coverage were applied to the calls made. The high fidelity of the mutations identified by using this approach is confirmed by the high proportion of calls with the C>* signature in a TpC context at all levels of allele fraction (Figure S1A).

Phasing Somatic Mutations with Heterozygous Germline SNPs

To determine the fraction of tumor cells carrying a given mutation, we use the following formula:

$$f = \min\left(1, \frac{r}{R}\frac{\rho\eta_T + (1-\rho)\eta_N}{\rho}\right),$$

where ρ is the fraction of cells in the sample that are tumor cells (derived here from the ASCAT package [Van Loo et al., 2010]); r is the number of reads reporting the variant allele out of R total reads across the base in question; and η_T and η_N are the copy number of the genome at that base in the tumor and normal genomes respectively.

For chromosomes showing subclonal copy number variation, we identified and phased mutations linked to nearby heterozygous germline SNPs. First, we identified heterozygous germline SNPs that were less than the maximum insert size away from a given mutation on the relevant chromosome (700bp in this sample). We then screened the sequencing file for read pairs where sequence covered both the SNP and the mutation, allowing us to determine which of the heterozygous SNP alleles was linked to the mutation. We determined whether that particular allele was on the deleted or retained copy of the chromosome by evaluating the number of reads from the tumor sample reporting either allele of the heterozygous SNP. If the observed fraction of reads reporting the germline allele linked to the mutation was significantly less than the expected ratio for the baseline state (ie 0.5 in a near-diploid chromosome) by a binomial test, we classified the mutation as being on the subclonally deleted chromosome. Where significantly greater than the expected ratio, we classified it as being on the retained copy. Where the observed fraction was not significantly different from the expected ratio, we identified the allele as part of the wider imputed haplotype (as discussed below) and determined whether the mutation was on the subclonally deleted or retained parental copy accordingly.

Phasing Two Nearby Subclonal Somatic Mutations

To identify pairs of subclonal mutations that were either mutually exclusive or showed subclonal evolution, we compiled a list of mutations present in < 100% of tumor cells and identified pairs of such mutations within an insert size of one another. In order to characterize a pair of subclonal mutations as showing subclonal evolution, we needed to find at least one example of a read pair reporting both variant alleles simultaneously AND one read pair reporting one variant allele but the wild-type allele for the other

locus. In addition, we required that no reads were seen reporting the reverse situation (wild-type for the former locus and variant for the latter locus).

To classify a mutation as "mutually exclusive," we could only examine regions for which the copy number in the tumor is 1. This is because for regions of higher copy number, mutations could be in the same subclone but on different parental copies of the chromosome. Within a region of copy number 1, the requirement for a mutually exclusive pair of mutations was that at least one read pair reported the variant allele at the 5′ locus and the wild-type allele at the 3′ locus AND one read pair reporting the wild-type allele at the 5′ locus and the variant allele at the 3′ locus. In addition, we required that no read pairs reported both variant alleles simultaneously.

Modeling Subclonal Structure Using Bayesian Dirichlet Processes

We model somatic mutations within the tumor as deriving from an unknown number of subclones, each of which is present at an unknown fraction of tumor cells and contributes an unknown proportion of all somatic mutations, with all the unknown parameters to be jointly estimated. We initially considered conventional mixture models, but found the requirement to specify the number of clusters too restrictive. For this reason, we used a Bayesian Dirichlet process (Dunson, 2010) to model the data.

Essentially, for a set of observed somatic mutations, we know the total read-depth across the base and the number of those reads reporting the variant allele for each mutation. We also know the expected fraction of reads that would report a mutation if present at one copy in 100% of tumor cells given the copy number at that locus and the normal cell contamination (through the equation shown above). Then,

$$y_i \sim \text{Bin}(N_i, \zeta_i \pi_i), \text{ with } \pi_i \sim DP(\alpha P_0),$$

where y_i is the number of reads reporting the ith mutation from N_i reads and ζ_i is the expected fraction of reads that would report a mutation present in 100% of tumor cells at that locus. Here, $\pi_i \in (0, 1)$, the fraction of tumor cells carrying the ith mutation, is modeled as coming from a Dirichlet process.

We use the stick-breaking representation of the Dirichlet process:

$$P = \sum_{h=1}^{\infty} \omega_h \delta_{\pi_h}, \text{ with } \pi_h \sim P_0,$$

where ω_h is the weight of the hth mutation cluster (that is, effectively the proportion of all somatic mutations specific to that cluster) and δ_π is a point mass at π. To capture the stick-breaking formulation, we let

$$\kappa_h = V_h \prod_{l<h} (1 - V_l), \text{ with } V_h \sim \text{Beta}(1, \alpha).$$

The complicating factor here is that for subclones representing a lower fraction of tumor cells (lower values of π_h), we have a lower sensitivity for identifying mutations in the original genome sequencing. We therefore correct the κ_h for the sensitivity S_π (probability of calling a mutation found in a fraction, θ, of tumor cells):

$$\omega_h = \frac{\kappa_h S_{\pi_h}}{\sum_i \kappa_i S_{\pi_i}}.$$

We assume S_π is known and calculable for all values of $\pi \in (0, 1)$. For the genomes described here, we use a three-parameter logistic curve where parameters are estimated from bootstrapped resamples by nonlinear least-squares (see below).

For the prior distributions, we let $P_0 \sim U(0, 1)$ and $\alpha \sim \Gamma(0.01, 0.01)$. We set a practical upper limit on h of 30. We find little difference in the model output if we vary these priors, and in particular that on α, even to the extent of fixing it at 1 (data not shown).

We use Gibbs sampling to estimate the posterior distribution of the parameters of interest, implemented in OpenBUGS v3.2.1. The code for this implementation is available with this paper (Supplemental Data). The Markov chain was run for 20,000 iterations, of which the first 13,000 were discarded. The chains converged rapidly to a stable limiting distribution, as assessed by using standard procedures. To generate the marginal distribution of subclonal mutations (such as those in Figure 1C and Figure 6B), both κ_h and π_h were monitored. The median and 95% posterior intervals of the density were estimated from π_h, each weighted by the associated value of κ_h, by using a Gaussian kernel (in R v2.13).

We first tested the Bayesian Dirichlet model on simulated data. To ensure the simulations replicated real data, the simulations used parameter estimates and sequence coverage matched to the values generated for PD4109a. The sensitivity for detecting mutations at different levels of subclonality in this sample is shown in Figure S6A—the parameter estimates for the logistic curve were then used to generate the "observed" mutations in the simulation. For each mutation, the read depth used for simulating the 232 mutations for the Dirichlet process was then based on the same read depth as for the 232 mutations confirmed by 454 sequencing in PD4109a. In the first simulation (Figures S1D and S1E), we set the "true" underlying make-up of the cancer to have 20% of the somatic mutations in all tumor cells and 80% mutations derived from three subclones (at 20%, 30%, and 60% of tumor cells; brown bars). Then, allowing

for variable sensitivity for detecting mutations at different fractions of tumor cells, we simulated a set of "observed" mutations from the cancer (blue bars). From the simulated set of observed mutations, we run the Dirichlet process model, which returns a distribution (green curves) very close to the "true" underlying distribution. In a second simulation, we assumed a completely different true underlying distribution: 40 subclones, each representing 2.5% of mutations and evenly spread through (0,1). Again, the Dirichlet process accurately modeled the "true" distribution (Figure S1D).

Estimating Sensitivity of Sequencing for Finding Subclonal Mutations

To model the sensitivity of the original genome sequencing for detecting mutations at different fractions of tumor cells, we undertook bootstrapping. We sampled 10,000 random wild-type positions in the genome for each level of subclonality, θ_h. At each of these random positions, we introduced a "subclonal mutation" in the following way. First, from the copy number of the locus, the normal cell contamination and the current level of subclonality θ_h, we calculated $\zeta_i\theta_h$, the fraction of reads expected to report the variant allele (by using the first formula in Extended Experimental Procedures). We choose the variant allele from $(A, C, G, T) \setminus$ (Reference allele). Then, for each of the reads from the tumor genome across this base, we draw from the U(0,1) distribution. If the draw is greater than $\zeta_i\theta_h$, we leave the base call unchanged. If the draw is less than $\zeta_i\theta_h$, and the base call = Reference allele, we change the base call to the variant allele. If the draw is less than $\zeta_i\theta_h$ and the base call is not the reference allele or the variant allele (i.e., there was a sequencing error), we leave it unchanged. If the draw is less than $\zeta_i\theta_h$ and the base call was the variant allele, we change it to one of the other three (nonvariant allele) bases. The reads from the normal genome we leave unchanged.

Thus, we generate a set of simulated mutations at each level of subclonality that exactly match the sequence coverage, sequencing error profiles, normal cell contamination and ploidy variation across the genome as seen in the original sequencing data. On the simulated sets of mutations, we run our substitution caller, Caveman, and count the number of mutations called at each level of subclonality.

From these bootstrapping estimates of sensitivity, we fit a three parameter logistic curve by using nonlinear least-squares estimation:

$$S_\theta = \frac{A}{1 + \exp((\chi - \log\theta)/\sigma)}.$$

Identifying Regions of Subclonal Copy Number Variation

Major and minor allele frequencies are estimated as the proportion of reads corresponding to each allele and therefore have binomial distributions. The observed distributions of the minor and major allele frequencies are distinct when the underlying allele frequencies are very different and when the read depth is high. In such regions, accurate estimates of the B-Allele Frequency (BAF) may be obtained by assuming that the allele frequencies above and below 0.5 arise from the two different haplotypes. However, as the BAF approaches 0.5 (as occurs in the case of subclonal aberrations) the two distributions increasingly overlap, resulting in wider confidence intervals in the estimated BAF. In order to separate the two distributions, we phased the observed genotypes by using Impute2 (Howie et al., 2009). Impute2 uses a set of polymorphic sites for which a reference panel of known genotypes is available. In this work we used the interim release from phase 1 of the 1000 Genomes Project, released in June 2011, as a reference panel (1000 Genomes Project Consortium, 2010). In the first step, the observed genotype of the matched normal samples at each of the polymorphic sites was identified. Impute2 was then run across these genotypes for the whole genome, in 5Mb segments, yielding phased haplotypes. Using the phased haplotypes, minor and major allele frequencies may be assigned to the two haplotypes. Within haplotype blocks of length ~300 kb the resulting haplotype frequencies lie in two separate bands for regions with subclonal or fully clonal copy number aberrations (CNAs). However, these blocks are separated by recombination hotspots which lead to "switching" of the blocks and hence the Battenberg pattern illustrated in Figure 2A (chromosome 7). For chromosomes or regions containing no CNA, the two distributions lie on top of each other, as illustrated in Figure 2A (chromosome 3).

In regions with a separation of haplotype frequency bands, the "switch-points" between consecutive haplotype blocks are clearly visible and can be straightforwardly detected by segmentation methods. We use segmentation by the Piecewise Constant Fitting (PCF) algorithm (with a breakpoint penalty parameter of 3) (Baumbusch et al., 2008) to determine "switch-points" of haplotype blocks. This approach allows haplotype phasing across large chromosomal distances (up to whole chromosomes, see Figure 2A) in regions of copy number imbalance.

Building on these long-range phased haplotype frequencies, we can discern both clonal and subclonal copy-number imbalances. The haplotype frequencies of clonal copy number changes must obey:

$$h_f = \frac{1 - \rho + \rho n_B}{2(1 - \rho) + \rho(n_A + n_B)}$$

where ρ is the fraction of tumor cells within the samples, and n_A and n_B are the (integer) allele-specific copy numbers of that locus. We obtain initial estimates of ρ (and the tumor ploidy ψ) from the ASCAT package (Van Loo et al., 2010) applied to Affymetrix SNP 6.0 array data, and further fine-tune these two-digit estimates to three-four digit estimates by applying the above equation to a large clonal

reference region with constant allele-specific copy number. To obtain allele-specific copy number estimates, we combine LogR data l_R (derived from local NGS read depth) with the haplotype frequency equation above, and build upon the model for allele-specific copy number derivation described previously (Van Loo et al., 2010):

$$n_A = \frac{\rho - 1 + (1 - h_f)\psi 2^{l_R}}{\rho}$$

$$n_B = \frac{\rho - 1 + h_f \psi 2^{l_R}}{\rho}.$$

As our haplotype frequency data show very little systematic bias (Figure 2A), and a significant part of LogR data bias is removed by GC wave correction, it is reasonable to assume that the error margin on these copy number estimates is significantly less than ± 1 for most genomic segments (with perhaps the exception of some highly amplified regions). Hence, under a model of clonality of a genomic segment under study, the haplotype frequencies of the germline heterozygous SNPs within that segment should be distributed around one of the four following values: $(1 - \rho + \rho\lfloor n_B \rfloor)/(2(1 - \rho) + \rho(\lfloor n_A \rfloor + \lfloor n_B \rfloor))$, $(1 - \rho + \rho\lfloor n_B \rfloor)/(2(1 - \rho) + \rho(\lfloor n_A \rfloor + \lfloor n_B \rfloor))$, $(1 - \rho + \rho\lceil n_B \rceil)/(2(1 - \rho) + \rho(\lfloor n_A \rfloor + \lceil n_B \rceil))$ or $(1 - \rho + \rho\lceil n_B \rceil)/(2(1 - \rho) + \rho(\lceil n_A \rceil + \lceil n_B \rceil))$. This framework provides the means to determine statistically if a copy number aberration is clonal or subclonal by a simple t test. We employ a two-sided t test with a = 0.05 and require a minimum deviation of 0.01 of segmented haplotype frequencies from their theoretical clonal states to call a segment subclonal.

For subclonal copy number changes, we aim to call copy number states in each of the subclones, and to quantify the cell percentages of each of these subclones. We base our approach on the assumption that any genomic aberration occurred only once during tumor evolution. Under this assumption, the haplotype frequency and coverage depth/LogR data of this genomic segment are shaped by three populations of cells: a fraction 1-ρ of admixed normal cells, a fraction $\rho\tau$ of tumor cells with the subclonal aberration and a fraction $\rho(1-\tau)$ of tumor cells without the subclonal aberration (with t $\in$ [0,1]). Note that these populations of tumor cells may each consist of one or multiple subclones.

To estimate τ, the fraction of tumor cells with the specific subclonal copy number change, we further assume that the subclonal copy number change resulted in a gain or a loss of exactly one copy of one allele. Given an error margin smaller than ± 1 on the allele-specific copy number estimates n_A and n_B, this assumption restrains the allele-specific copy number states in both subclones to one of four combinations: (i) $\lfloor n_A \rfloor + \lfloor n_B \rfloor$ in one subclonal cell population and $\lceil n_A \rceil + \lfloor n_B \rfloor$ in the other subclonal cell population, (ii) $\lfloor n_A \rfloor + \lfloor n_B \rfloor$ and $\lfloor n_A \rfloor + \lceil n_B \rceil$, (iii) $\lceil n_A \rceil + \lfloor n_B \rfloor$ and $\lceil n_A \rceil + \lceil n_B \rceil$ or (iv) $\lfloor n_A \rfloor + \lceil n_B \rceil$ and $\lceil n_A \rceil + \lceil n_B \rceil$. For any specific haplotype frequency h_f of a genomic segment (that as detailed above shows very little bias), only two of these combinations are possible (i.e., a line with constant h_f drawn in allele-specific copy number space will intersect the square defined by vertices $(\lfloor n_A \rfloor, \lfloor n_B \rfloor)$, $(\lceil n_A \rceil, \lfloor n_B \rfloor)$, $(\lfloor n_A \rfloor, \lceil n_B \rceil)$ and $(\lceil n_A \rceil, \lceil n_B \rceil)$ exactly twice in the case of a subclonal aberration). It can be shown that one of these combinations is always a mix of states with total copy number $\lfloor n_A \rfloor + \lfloor n_B \rfloor$ and $\lfloor n_A \rfloor + \lfloor n_B \rfloor + 1$, whereas the other is always a mix of states with total copy number $\lfloor n_A \rfloor + \lfloor n_B \rfloor + 1$ and $\lfloor n_A \rfloor + \lfloor n_B \rfloor + 2$ (i.e., the line with constant h_f in allele-specific copy number space has a positive slope). Therefore, the total copy number $n_A + n_B$, or the value of l_R (as $n_A + n_B = (2\rho - 2 + \psi 2^{l_R})/\rho$) can be used to select the final combination of states. Given that combination of allele-specific copy number states ($n_{A,1}$, $n_{B,1}$) in a fraction of tumor cells τ and ($n_{A,2}$, $n_{B,2}$) in a fraction of tumor cells 1-τ, the haplotype fraction h_f of the segment is given by:

$$h_f = \frac{1 - \rho + \rho\tau n_{B,1} + \rho(1 - \tau) n_{B,2}}{2 - 2\rho + \rho\tau(n_{A,1} + n_{B,1}) + \rho(1 - \tau)(n_{A,2} + n_{B,2})}$$

Hence, τ can be calculated as:

$$\tau = \frac{1 - \rho + \rho n_{B,2} - 2h_f(1 - \rho) - h_f\rho(n_{A,2} + n_{B,2})}{h_f\rho(n_{A,1} + n_{B,1}) - h_f\rho(n_{A,2} + n_{B,2}) - \rho n_{B,1} + \rho n_{B,2}}$$

Finally, standard deviations and 95% confidence intervals can be calculated for this value by using a bootstrapping approach (for each segment, allele frequencies are resampled 1,000 times, with replacement).

SUPPLEMENTAL REFERENCES

1000 Genomes Project Consortium. (2010). A map of human genome variation from population-scale sequencing. Nature *467*, 1061–1073.

Baumbusch, L.O., Aarøe, J., Johansen, F.E., Hicks, J., Sun, H., Bruhn, L., Gunderson, K., Naume, B., Kristensen, V.N., Liestøl, K., et al. (2008). Comparison of the Agilent, ROMA/NimbleGen and Illumina platforms for classification of copy number alterations in human breast tumors. BMC Genomics *9*, 379.

Dunson, D.B. (2010). Nonparametric Bayes applications to biostatistics. In Bayesian Nonparametrics, N.L. Hjort, C. Holmes, P. Müller, and S.G. Walker, eds. (Cambridge: Cambridge University Press).

Greenman, C.D., Pleasance, E.D., Newman, S., Yang, F., Fu, B., Nik-Zainal, S., Jones, D., Lau, K.W., Carter, N., Edwards, P.A., et al. (2012). Estimation of rearrangement phylogeny for cancer genomes. Genome Res. *22*, 346–361.

Howie, B.N., Donnelly, P., and Marchini, J. (2009). A flexible and accurate genotype imputation method for the next generation of genome-wide association studies. PLoS Genet. *5*, e1000529.

Van Loo, P., Nordgard, S.H., Lingjærde, O.C., Russnes, H.G., Rye, I.H., Sun, W., Weigman, V.J., Marynen, P., Zetterberg, A., Naume, B., et al. (2010). Allele-specific copy number analysis of tumors. Proc. Natl. Acad. Sci. USA *107*, 16910–16915.

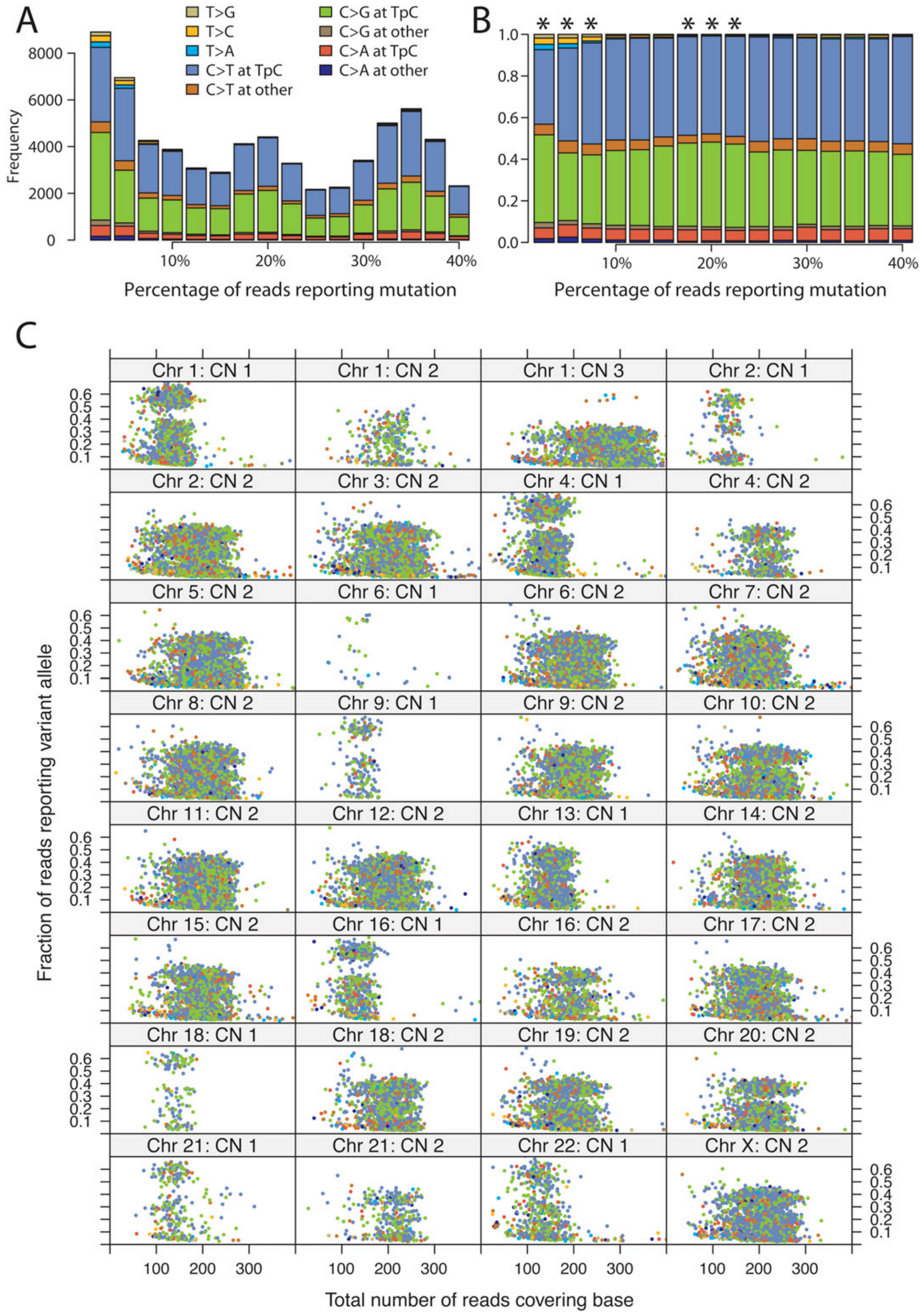

Figure S1. Subclonal Mutations in PD4120a, Related to Figure 1

(A) Observed distribution of mutation signatures for different values of the variant allele fraction, showing that even with rare mutations, the C>* signature in a TpC context is preserved.

(B) Observed fraction of mutation signatures for different values of the variant allele fraction. Those levels of variant allele fraction that show a significantly different distribution from the distribution of fully clonal mutations are marked with an asterisk (*).

(C) Lattice plot showing the distribution of mutations separately for each copy number segment in the PD4120a genome. The *x* axis denotes the total number of reads covering the mutations and the *y* axis the variant allele fraction. The points are colored according to the spectrum of mutations, by using the key shown for (A).

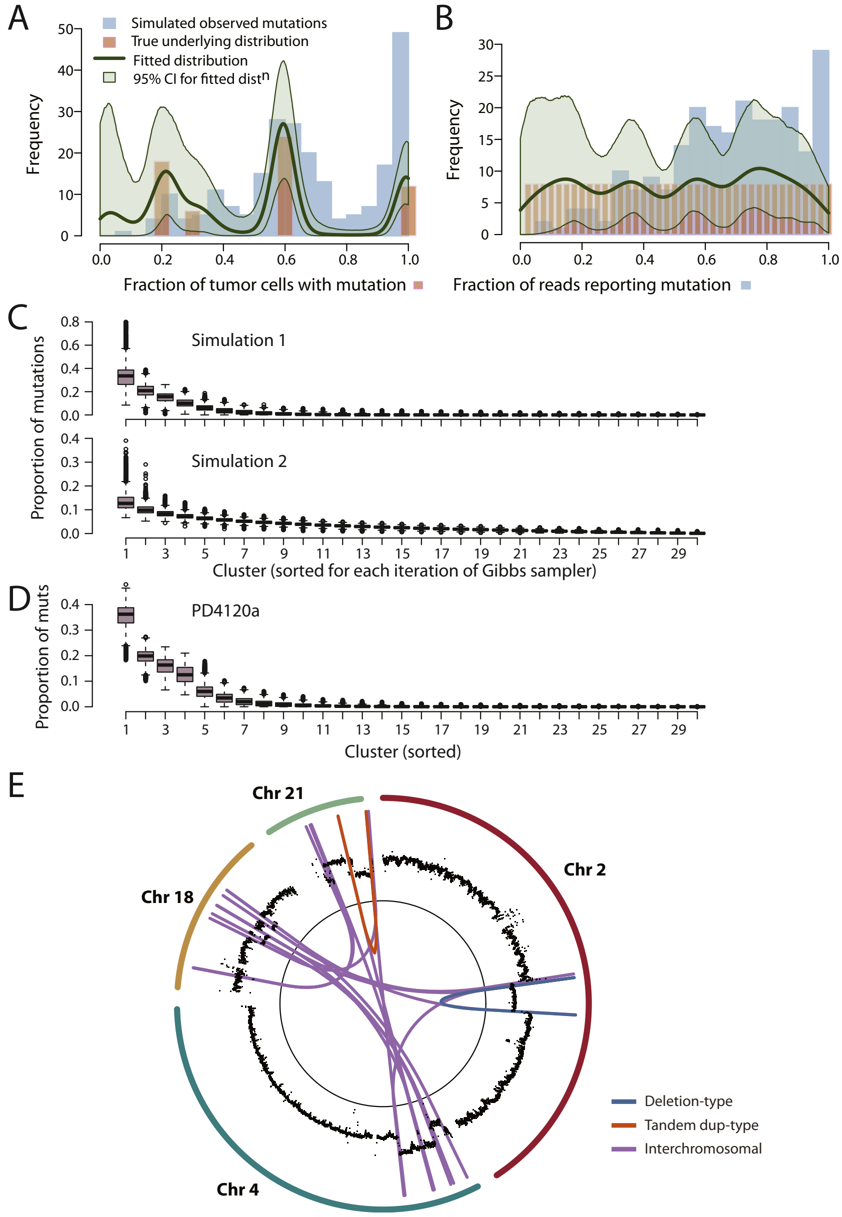

Figure S2. Modeling Clusters of Subclonal Mutations, Related to Figure 1

(A) Mutations (blue histogram) from an *in silico* simulation of a tumor in which fully clonal mutations account for 20% of mutations, 40% mutations are found in a subclone representing 60% of tumor cells, 10% mutations in a subclone at 30% and 20% mutations in a subclone at 20% of tumor cells (pink bars). The simulated mutations have also been subject to correction for the sensitivity of detection at different fractions of tumor cells, hence there are fewer "observed" mutations at 20% of tumor cells than at 100% despite there being more "true" mutations at this level. Statistical modeling by a Bayesian Dirichlet process of the simulated mutations is shown as a dark green line. Also shown are the 95% posterior confidence intervals for the fitted distribution (pale green area).

(B) Mutations (blue histogram) from an *in silico* simulation of a tumor in which there are 40 subclones, evenly spread from 0%–100% of tumor cells and each contributing 2.5% of mutations (pink bars). The simulated mutations have been subject to correction for the sensitivity of detection at different fractions of tumor cells, hence there are fewer "observed" mutations at 20% of tumor cells than at 100% despite there being the same number of "true" mutations at this level. Statistical modeling by a Bayesian Dirichlet process of the simulated mutations is shown as a dark green line. Also shown are the 95% posterior confidence intervals for the fitted distribution (pale green area).

(C) Box and whisker plots showing the posterior distributions for the weights of each of the 30 clusters, ordered from greatest to least, for the two simulations shown in (D) and (E). The first simulation, based on four subclones, shows nonnegligible weights for the first 4–5 subclones, but rapidly tails to 0 thereafter. For the second simulation, based on 40 subclones, all at constant weight, the distribution of weights is much flatter, and does not hit 0 until beyond 15–20 clusters.

(D) Box and whisker plots showing the posterior distributions for the weights of each of the 30 clusters, ordered from greatest to least, for PD4120a. The first 4–5 clusters show nonnegligible weights, but they tail rapidly to 0 thereafter.

(E) Circle plot showing the copy number (black points) and rearrangements for a chromothripsis event involving chromosomes 2, 4, 18, and 21.

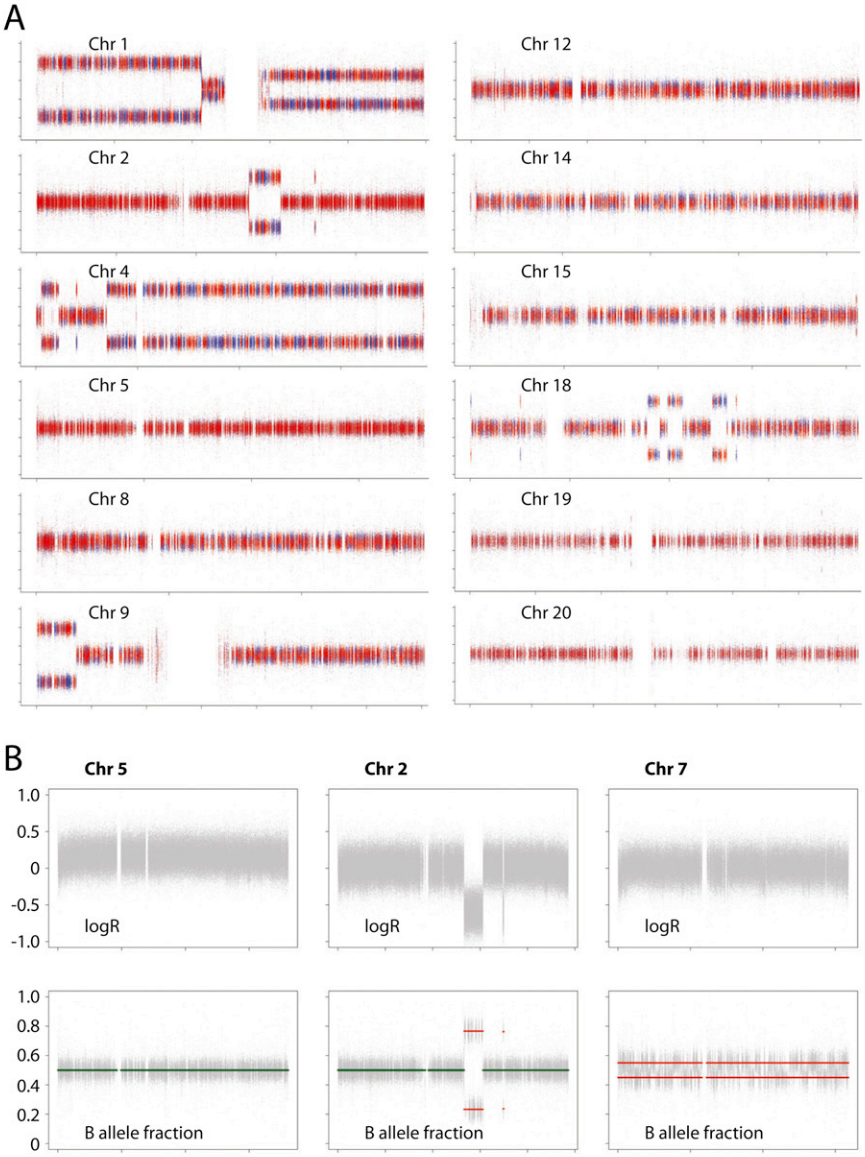

Figure S3. Subclonal copy number variation in PD4120a, Related to Figure 2

(A) Battenberg plots of allele fractions for phased parental haplotypes for various chromosomes. Each heterozygous germline SNP is phased into two possible parental states by imputation. The observed allele fraction for one phased chromosomal copy is plotted in blue and the other in red. For a chromosome, such as chromosome 5, showing no subclonal copy number variation, both parental copies are present at exactly equal proportions and the red and blue points are superimposed around an allele fraction of 0.5. For chromosomes showing subclonal copy number variation, such as chromosome 8 and chromosome 13, the parental copies are present at unequal ratios, leading to separation between the red and blue segments. The extent of separation is correlated with the fraction of tumor cells showing the chromosomal gain or loss.

(B) Copy number profiles for logR and B allele fraction for chromosomes 2, 5, and 7 of PD4120a. Note that the logR value for chromosome 2 is virtually the same as for chromosome 7 and substantially lower than that for chromosome 5, indicating that, like chromosome 7, chromosome 2 is deleted in a sizable subclone of cells. However, the B allele fraction for chromosome 2 is exactly balanced at 0.5, implying that both parental copies are deleted in equal proportions.

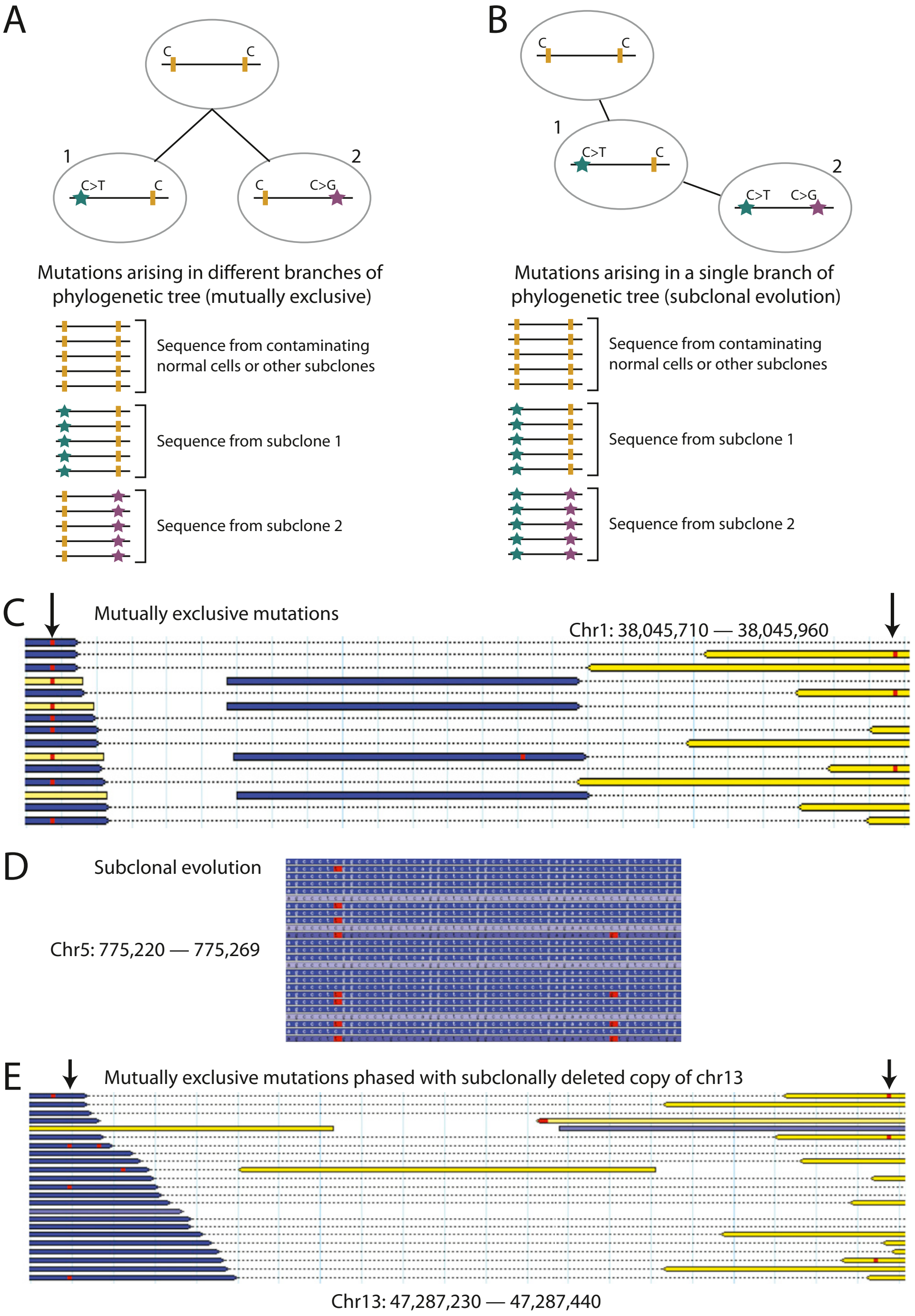

Figure S4. Phasing Pairs of Subclonal Point Mutations, Related to Figure 3

(A) Phasing of subclonal mutations (stars) with other nearby subclonal mutations allows determination of whether they are in separate phylogenetic lineages, in which case no sequencing reads will report both variants together (mutually exclusive pair of mutations).

(B) Similar phasing analysis can identify cases where the later subclonal mutation has arisen on an allele linked with a previous subclonal mutation.

(C) Example of a mutually exclusive pair of mutations from PD4120a. Sequencing read pairs are shown as yellow and blue bars linked by a dotted line. Base calls varying from the reference genome are shown as red squares. Two nearby mutations, indicated by arrows, are never found on the same read pair.

(D) Example of a pair of mutations showing subclonal evolution in PD4120a. The right-hand subclonal mutation occurred on an allele already carrying the left-hand mutation, as evidenced by the existence of reads reporting both together, the left-hand but not the right-hand mutation but never the right-hand mutation without the left-hand one.

(E) A pair of mutations, both of which phase with the subclonally deleted copy of chromosome 13, but are mutually exclusive.

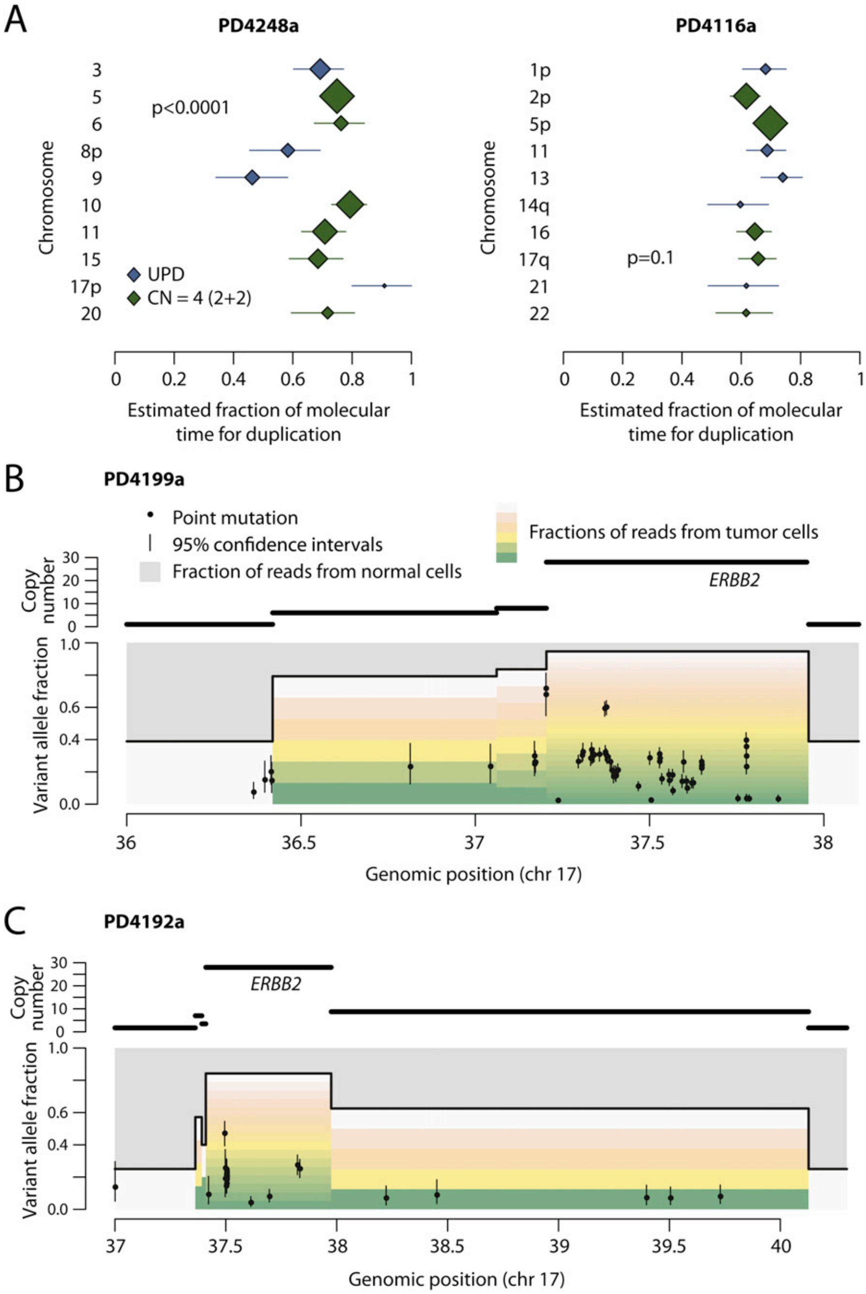

Figure S5. Timing of Chromosomal Gains and Genomic Amplifications, Related to Figure 4

(A) Forest plots showing the point estimates (diamonds) and 95% confidence intervals estimated by bootstrapping of particular chromosomes for two breast cancer genomes. The size of the diamond is proportional to the number of mutations considered, and the color by whether the chromosomal gain reflects uniparental disomy (blue) or tetraploid chromosomes (green). The estimates show significant heterogeneity for PD4248a ($p < 0.0001$) but not for PD4116a ($p = 0.3$), with the latter indicating the possibility of all the gains occurring as a single endoreduplication event.

(B and C) Timing of *ERBB2* genomic amplification for PD4199a (B) and PD4192a (C). Here, the top panel shows the copy number segments for the region of chromosome 17 around *ERBB2*. The lower panel shows the point mutations as black points, with the *x* axis reflecting the genomic position and the *y* axis the variant allele fraction. The 95% confidence intervals for the variant allele fraction are shown as vertical bars for each mutation. The allele fraction is divided by the colored bars into the proportions of reads derived from contaminating normal cells (gray bars) and the fraction coming from each of the copies of that segment in the tumor cells (the multiple bars from green to yellow to pink to white). Early mutations will be found relatively higher up these bars, whereas late ones will be seen toward the bottom of the variant allele fraction.

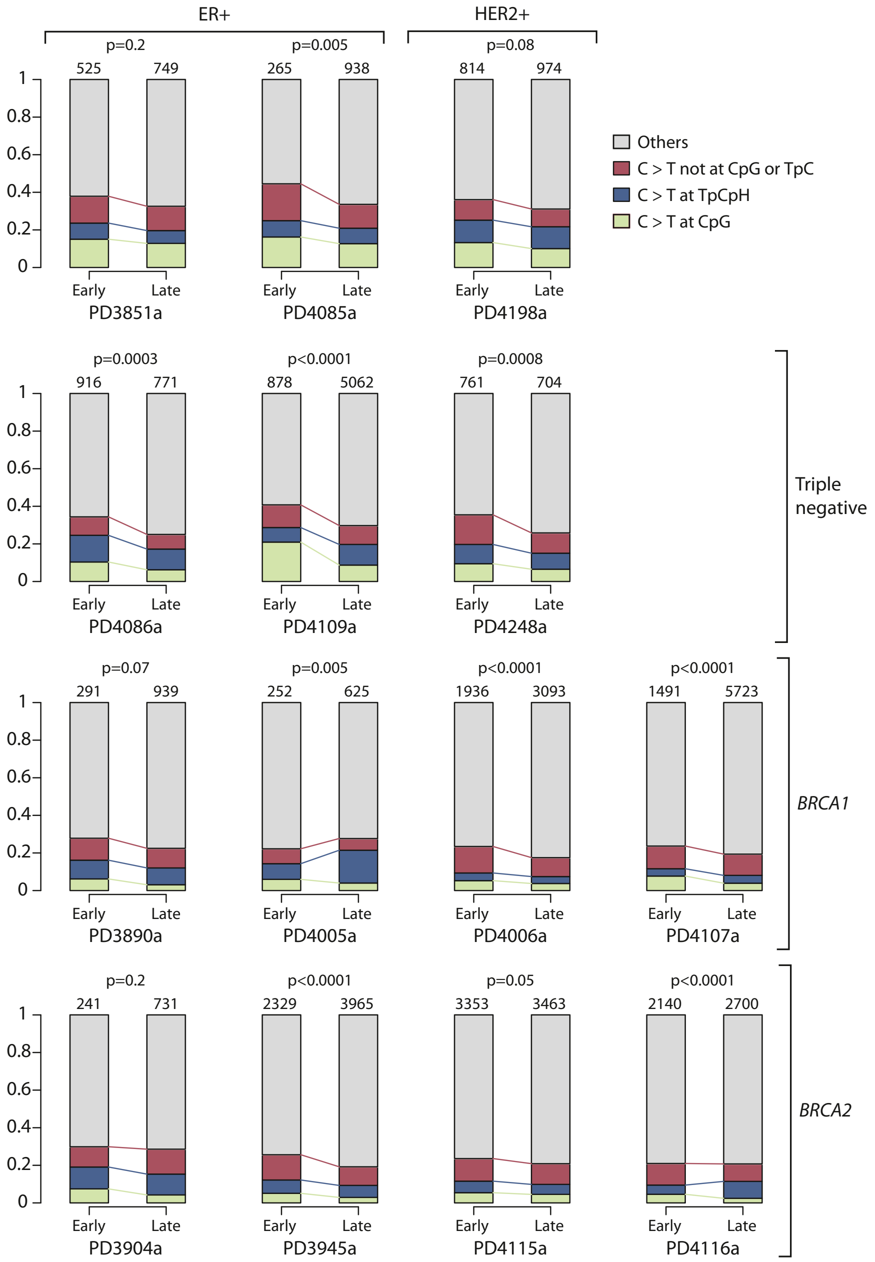

Figure S6. Comparison of Early and Late C>T Point Mutation Signatures in 14 Informative Breast Cancer Genomes, Related to Figure 5
Stacked bar charts showing the fraction of early mutations (ploidy > 1) and late mutations (ploidy = 1) accounted for by each mutation type. The p values refer to the overall difference in distribution between early and late C>T mutations (chi-square test). The numbers above each stacked bar denotes the number of early or late mutations analyzed.

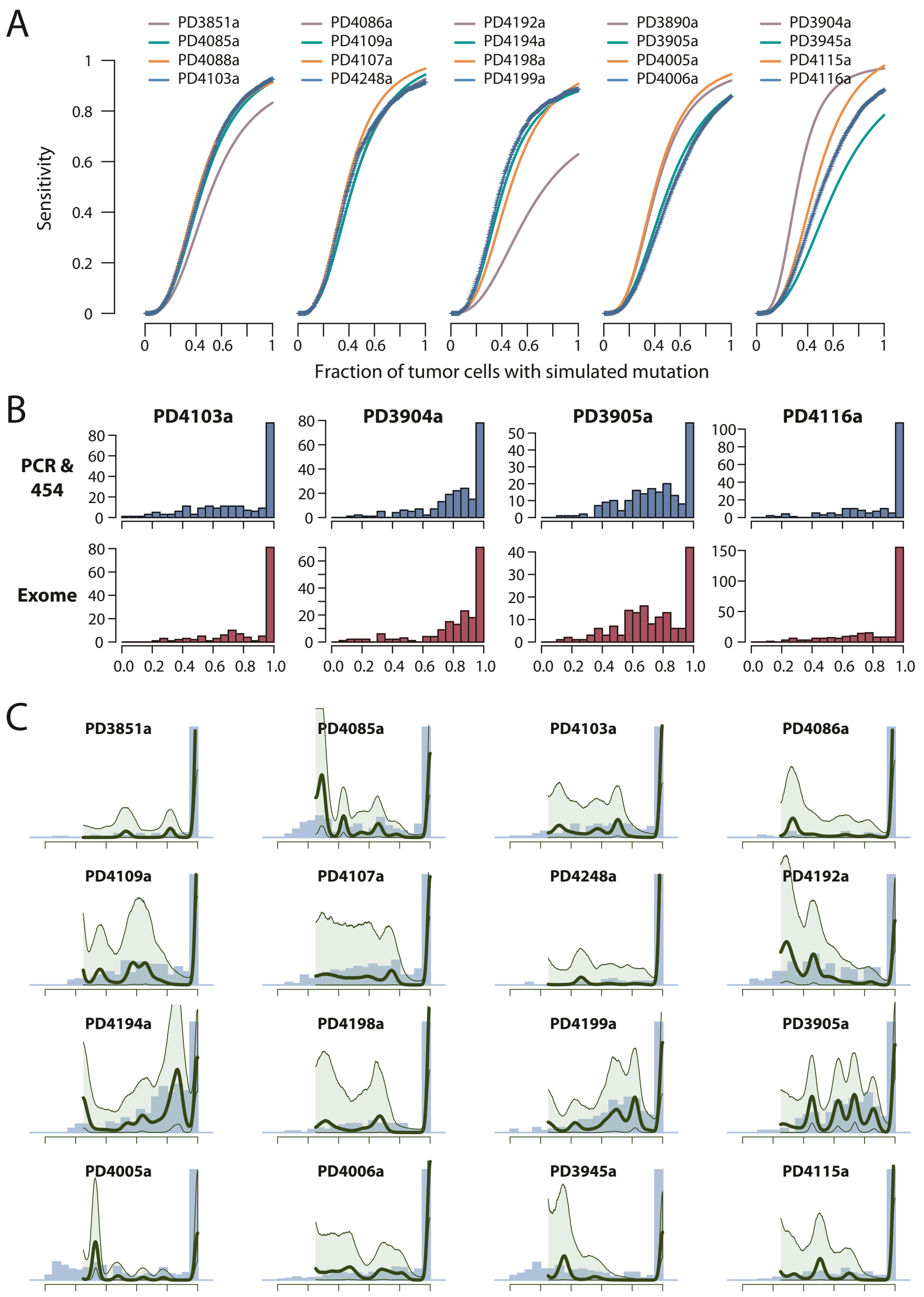

Figure S7. Patterns of Subclonal Mutation in 20 Breast Cancer Genomes, Related to Figure 6

(A) Fitted three-parameter logistic curves to bootstrapped estimates of sensitivity for mutations at different levels of subclonality derived from each of the 20 breast cancer genomes. For the five samples colored blue the raw bootstrapped values are shown (as plus [+] symbols), to allow assessment of goodness-of-fit of the logistic curve to the raw data.

(B) Comparison of the empiric distributions of subclonal mutations between PCR with deep pyrosequencing on the 454 platform and exome pull-down and sequencing for four patients. For each histogram, point mutations called in the original whole-genome sequencing were identified for which there was independent validation by either 454 sequencing or exome pull-down. The distributions of subclonality obtained from each validation method are then plotted in the relevant histogram.

(C) Statistical modeling by a Bayesian Dirichlet process of the distribution of clonal and subclonal mutations for 16 breast cancers. The empiric histogram of mutations is shown in pale blue, with the fitted distribution as a dark green line. Also shown are the 95% posterior confidence intervals for the fitted distribution (pale green area).

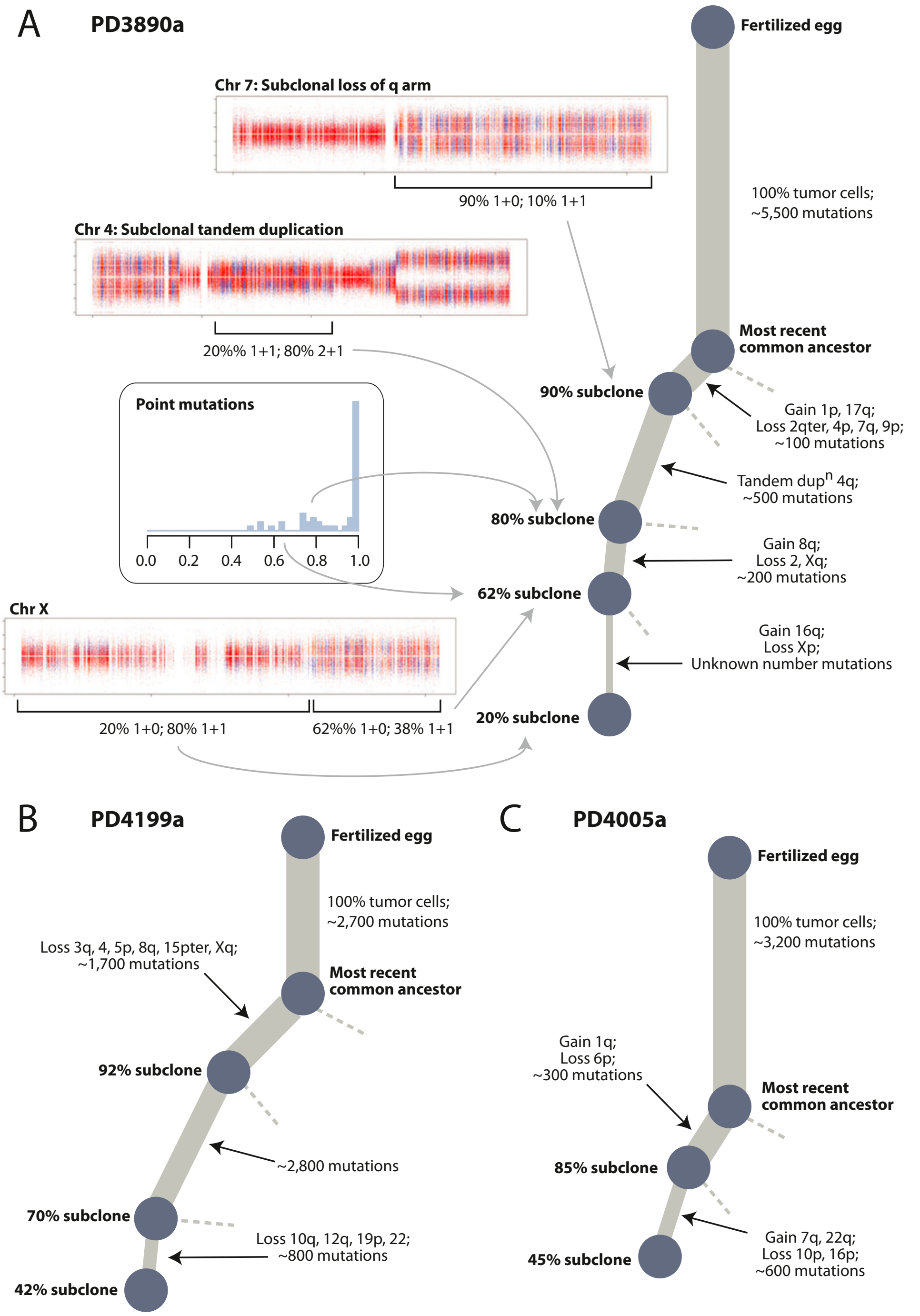

Figure S8. Phylogenetic Trees for Three Breast Cancer Patients, Linked to Figure 7

(A) Phylogenetic tree and supporting data for PD3890a. The Battenberg plot for chromosome 7 shows evidence that the q arm shows loss of heterozygosity (LOH; 1+0) in 90% of tumor cells, with normal diploidy (1+1) in 10%. Because LOH is a one-directional "valve" (once lost, heterozygosity cannot be regained), it follows that diploidy is the ancestral state and LOH is the derived state. Hence, this is direct evidence of a subclone representing 90% of tumor cells, also carrying several other copy number changes and approximately 100 point mutations. The centromeric portion of 4q shows evidence for a mix of 2+1 copies in 80% of tumor cells and normal diploidy (1+1) in 20% of tumor cells. From the rearrangement data, this copy number gain is caused by a subclonal tandem duplication, implying that the 2+1 copy number state found in 80% tumor cells is the derived state. This indicates the existence of an 80% subclone, matched by a small cluster of point mutations seen on sequencing of the exome (inset). Finally, chromosome X, among others, shows evidence for a 62% subclone and 20% subclone. By repeated application of the pigeonhole principle, each of these subclones must be collinear on the phylogenetic tree.

(B) Phylogenetic tree for PD4199a.

(C) Phylogenetic tree for PD4005a.
